# Distinct role of mitochondrial function and protein kinase C in intimal and medial calcification *in vitro*

**DOI:** 10.3389/fcvm.2022.959457

**Published:** 2022-09-20

**Authors:** Marina A. Heuschkel, Anne Babler, Jonas Heyn, Emiel P. C. van der Vorst, Marja Steenman, Maren Gesper, Ben A. Kappel, David Magne, Yann Gouëffic, Rafael Kramann, Willi Jahnen-Dechent, Nikolaus Marx, Thibaut Quillard, Claudia Goettsch

**Affiliations:** ^1^Department of Internal Medicine I–Cardiology, Medical Faculty, RWTH Aachen University, Aachen, Germany; ^2^Institute of Experimental Medicine and Systems Biology, University Hospital, RWTH Aachen, Aachen, Germany; ^3^Interdisciplinary Center for Clinical Research, Institute for Molecular Cardiovascular Research, RWTH Aachen University, Aachen, Germany; ^4^Department of Pathology, Cardiovascular Research Institute Maastricht, Maastricht University Medical Centre, Maastricht, Netherlands; ^5^Institute for Cardiovascular Prevention (IPEK), Ludwig-Maximilians-University Munich, Munich, Germany; ^6^DZHK (German Centre for Cardiovascular Research), Partner Site Munich Heart Alliance, Munich, Germany; ^7^L’institut Du Thorax, Inserm UMR 1087, CNRS, INSERM, France and Nantes Université, Nantes, France; ^8^ICBMS UMR CNRS 5246, Université Claude Bernard Lyon 1, Villeurbanne, France; ^9^Department of Vascular Surgery, Vascular Center, Groupe Hospitalier Paris Saint-Joseph, Paris, France; ^10^Department of Nephrology and Clinical Immunology, University Hospital RWTH Aachen, Aachen, Germany; ^11^Department of Internal Medicine, Nephrology and Transplantation, Erasmus Medical Center, Rotterdam, Netherlands; ^12^Biointerface Laboratory, Helmholtz Institute for Biomedical Engineering, RWTH Aachen University, Aachen, Germany; ^13^PHY-OS Laboratory, INSERM UMR 1238, Nantes University of Medicine, Nantes, France

**Keywords:** vascular calcification, vascular smooth muscle cells, drug repurposing, mitochondrial function, matrix mineralization, protein kinase C

## Abstract

**Introduction:**

Vascular calcification (VC) is a major risk factor for cardiovascular morbidity and mortality. Depending on the location of mineral deposition within the arterial wall, VC is classified as intimal and medial calcification. Using *in vitro* mineralization assays, we developed protocols triggering both types of calcification in vascular smooth muscle cells (SMCs) following diverging molecular pathways.

**Materials and methods and results:**

Human coronary artery SMCs were cultured in osteogenic medium (OM) or high calcium phosphate medium (CaP) to induce a mineralized extracellular matrix. OM induces osteoblast-like differentiation of SMCs–a key process in intimal calcification during atherosclerotic plaque remodeling. CaP mimics hyperphosphatemia, associated with chronic kidney disease–a risk factor for medial calcification. Transcriptomic analysis revealed distinct gene expression profiles of OM and CaP-calcifying SMCs. OM and CaP-treated SMCs shared 107 differentially regulated genes related to SMC contraction and metabolism. Real-time extracellular efflux analysis demonstrated decreased mitochondrial respiration and glycolysis in CaP-treated SMCs compared to increased mitochondrial respiration without altered glycolysis in OM-treated SMCs. Subsequent kinome and *in silico* drug repurposing analysis (Connectivity Map) suggested a distinct role of protein kinase C (PKC). *In vitro* validation experiments demonstrated that the PKC activators prostratin and ingenol reduced calcification triggered by OM and promoted calcification triggered by CaP.

**Conclusion:**

Our direct comparison results of two *in vitro* calcification models strengthen previous observations of distinct intracellular mechanisms that trigger OM and CaP-induced SMC calcification *in vitro*. We found a differential role of PKC in OM and CaP-calcified SMCs providing new potential cellular and molecular targets for pharmacological intervention in VC. Our data suggest that the field should limit the generalization of results found in *in vitro* studies using different calcification protocols.

## Introduction

Cardiovascular diseases are the leading cause of death worldwide (1). Vascular calcification (VC) is a significant risk factor for cardiovascular morbidity and mortality in patients with end-stage renal disease, diabetes, and atherosclerosis (2). However, no pharmaceutical therapy is available to prevent or halt VC progression.

Based on the deposition site of minerals within the arterial wall, VC can be classified into two main types: intimal calcification observed in advanced atherosclerotic plaques and medial calcification lacking lipid deposits, most prevalent in end-stage renal disease and diabetes (3).

Historically, VC is regarded as a degenerative process including cell and tissue inflammation, degeneration, and remodeling, ultimately resulting in extracellular matrix (ECM) mineral deposition. Alternatively, calcification of both the tunica intima and tunica media is considered a cell-autonomous process reminiscent of osteogenesis (4). Vascular smooth muscle cells (SMCs) are the most abundant cell type in the arterial vessel wall and contribute to VC through osteochondrogenic transdifferentiation, characterized by the expression of osteogenic markers, elaboration of a mineralization competent extracellular matrix, and shedding of calcifying extracellular vesicles (3, 5).

Although both intimal and medial calcification results in ectopic calcification, it seems likely that they are triggered by different initiating and propagating molecular mechanisms (3). The variety of factors triggering VC development is reflected in various experimental *in vivo* and *in vitro* models (6). For example, osteogenic medium, commonly used to differentiate mesenchymal stem cells to osteoblasts, induces marked phenotypic changes of SMCs characterized by a loss of contractile markers and increased expression of bone-related genes. This mimics osteoblastogenesis in intimal calcification, a key process in atherosclerotic plaque remodeling (7, 8). On the other hand, a medium enriched in phosphate mimics the hyperphosphatemia associated with the pathophysiology of chronic kidney disease (CKD), which is a prevalent risk factor for medial calcification (9–11). Therefore, this study investigates and comparatively analyzes two different calcification protocols reflecting intimal and medial calcification. We hypothesize that distinct mineralization protocols alter specific intracellular mechanisms associated with SMC transdifferentiation and extracellular matrix mineralization.

## Materials and methods

### Human primary vascular smooth muscle cells

Human coronary artery SMCs (PromoCell, pSMCs) were grown in SMC growth medium 2 (SMC-GM2, PromoCell) supplemented with Smooth Muscle Cell Growth Medium 2 Supplement Mix (Promocell) consisting of 0.5 ng/ml epidermal growth factor, 5 μg/ml insulin, 2 ng/ml basic fibroblast growth factor, 1% penicillin/streptomycin (P/S), and 5% fetal bovine serum (FBS) at 37°C in humidified 5% CO_2_. Cells were used between passages 3 and 9 from at least three independent cell donors.

### Immortalized vascular smooth muscle cells

To generate immortalized SMC (iSMC) lines, primary human coronary artery SMCs were cultured in Dulbecco’s Modified Eagle Medium (DMEM, Thermo Scientific) with 4.5 g/L glucose, 10% FBS, 1% P/S at 37°C in humidified 5% CO_2_ (Thermo Fisher) and immortalized using SV40LT and HTERT. Retroviral particles were produced by transient transfection of HEK293T cells using TransIT-LT (Mirus). Two types of amphotropic particles were generated by co-transfection of plasmids pBABE-puro-SV40-LT (Addgene) or xlox-dNGFR-TERT (Addgene) in combination with a packaging plasmid pUMVC (Addgene) and a pseudotyping plasmid pMD2.G (Addgene). Retroviral particles were 100x concentrated using Retro-X concentrator (Clontech) 48 h post-transfection. Cell transduction was initiated by incubating the target cells with the retroviral supernatants for 48 h. After 7 days, the infected cells were selected with 2 μg/ml puromycin for 72 h.

### Calcification assays and visualization

pSMCs and iSMCs were cultured in the presence of either control medium (CM, DMEM, 10% FBS, 1% P/S), osteogenic medium [OM, consisting of CM supplemented with 10 nM dexamethasone (Sigma-Aldrich), 10 mM β-glycerol phosphate (Sigma-Aldrich) and 100 μM l-ascorbate phosphate (Sigma-Aldrich)], or CaP [consisting of CM supplemented with 1.8 mM CaCl_2_ (ROTH) and 0.9 mM Na_2_HPO_4_/NaH_2_PO_4_ (ROTH)] to reach a final concentration of 3 mM calcium and 2 mM phosphate. The concentrations of CaP aim to mimic the calcium and phosphate serum levels in CKD patients of 9.1 ± 0.7 mg/dL (2.27 mM) and 5.3 ± 1.4 mg/dL (1.72 mM) respectively (12). Media exchange was performed twice weekly.

Mineralized matrix formation was assessed by Alizarin Red S staining. Cell cultures were fixed with 4% paraformaldehyde (PFA) and stained with 2% (w/v) Alizarin Red S (pH 4.2, Sigma-Aldrich) for 30 min at room temperature. Excess dye was removed by washing with distilled water and imaged under a light microscope (EVOS^®^ FL Cell Imaging System). The staining was quantified by Alizarin Red S elution from the extracellular matrix using 100 mM cetylpyridinium chloride (Sigma-Aldrich) in water for 20 min at 37°C. Absorption was measured at 570 nm in a spectrophotometer (TECAN).

### Stimulation of vascular smooth muscle cells

Cells were stimulated with PD184352 (Sigma-Aldrich), prostratin (Sigma-Aldrich), ingenol,3,20-dibenzoate (Enzo LifeSciences), L690,330 (Tocris), fluticasone propionate (Sigma), or Go6983 (Tocris) dissolved in dimethylsulfoxide (DMSO) or water (for L690,330). An equal amount of the vehicle (1:1,000) was used as solvent control.

### Cell viability

Cell viability was assessed using the AlamarBlue assay (Thermo Scientific), according to the manufacturer’s protocol. Upon entering the viable cell, resazurin–the active compund of AlamarBlue–is reduced to resorufin that is assessed by fluorescence at Ex_560_ nm/Em_590_ nm.

Furthermore, cell viability was assessed by a live/dead fluorescence-based cell assay using fluorescein diacetate (FDA) and propidium iodide (PI). Staining of cells was performed using a mixture of 0.5 μg/ml FDA (Sigma-Aldrich) and (0.05 μg/ml PI), (Sigma-Aldrich) in PBS for 30 s. Cells treated with 0.5% Triton X-100 (Sigma-Aldrich) for 2 min served as a positive control for cell death. Following staining, cells were washed with PBS and examined by fluorescence microscopy. Image J v2.0 software was employed for quantification. Fluorescence images were converted into single-channel 8-bit grayscale images, and the threshold was adjusted to measure the mean gray values. FDA mean fluorescence intensity was divided by the corresponding PI mean fluorescence intensity to calculate the FDA/PI ratio at day 0 and day 7.

### Activity of tissue non-specific alkaline phosphatase

Tissue non-specific alkaline phosphatase (TNAP) activity was measured in cells using the Alkaline Phosphatase Activity Colorimetric Assay Kit (BioVision) according to the manufacturer’s protocol and normalized to the total protein amount assessed by bicinchoninic acid (BCA) assay (Thermo Scientific).

### Ribonucleic acid preparation and real-time polymerase chain reaction

Total RNA was isolated using TRIzol reagent (Life Technologies). Reverse transcription was performed using the High capacity cDNA Reverse Transcription Kit (Life Technologies), according to the manufacturer’s protocol. The gene expression levels were quantified by TaqMan-based real-time PCR reactions (Life Technologies). The used TaqMan probes are listed in Supplementary Table 1. The expression levels were normalized to RPLP0. Results were calculated using the ΔΔCt method and presented as fold increase relative to control.

### Gene expression analysis

300 ng of total RNA from calcifying and control pSMCs were processed using the GeneChip WT PLUS Reagent Kit (Affymetrix, Inc., Santa Clara, CA, United States) following the manufacturer’s protocol to yield purified biotinylated sense-stranded cDNA. Hybridization was performed to Clariom D Human Arrays using the GeneChip Hybridization, Wash & Stain Kit (Affymetrix, Inc., Santa Clara, CA, United States) and Fluidics Station 450 for 16 h at 45°C. Arrays were scanned using Affymetrix GeneChip Scanner 3000 controlled by GeneChip Command Console (AGCC) version 4.0 to produce CEL intensity files. The raw data were analyzed using the Transcriptome Analysis Console software (TAC4.0, ThermoFisher Scientific, United States) with default parameters for gene-level expression analysis based on the annotation Hg38 clariom_D_Human.r1.na36.hg38.a1.transcript.csv. SST-RMA was applied for normalization and summarization. Values are defined as log2 scaled normalized gene level expression values.

Microarray data have been deposited in NCBI’s GEO and are accessible through GEO Series accession number GSE211752.

Heatmaps and volcano plots were generated using the R statistical software environment. Heatmaps were visualized using the heatmap 0.2 function in the ggplot package and volcano plots were generated with the EnhancedVolcano package version 1.4.0 (13). The ConsensusPathDB database^1^ was used for pathway over-representation analysis, employing the canonical pathways from the Kyoto Encyclopedia of Genes and Genomes (KEGG) and Reactome. Pathways with a *p*-value < 0.05 were considered to be significantly enriched in a gene set of interest.

### Western blot analysis

Cells were lysed with RIPA buffer (Thermo Scientific) containing protease and phosphatase inhibitor (Roche). Protein concentration was measured using the BCA assay (Thermo Scientific) according to the manufacturer’s instructions. 15 μg protein was separated in 8% polyacrylamide gel, transferred to a nitrocellulose membrane, and incubated overnight with OXPHOS (1:1000, Abcam, ab110413) and human beta-actin (1:10.000; Sigma-Aldrich, A2228). Bound antibodies were then detected using HRP-conjugated secondary antibody (anti-mouse: #7076, Cell Signaling) and visualized by enhanced Super Signal West blotting substrate (ThermoFisher Scientific) with a ChemiDoc™ MP Imaging System and the software Image Lab version 6.0. Protein bands were quantified by FIJI (ImageJ) software (Version 1.53c), and normalized to the loading control beta-actin.

### Collagen contraction assay

1.5 × 10^5^ cells per mL were embedded in collagen gels from rat tail collagen type 1 (R&D system). 200 μL of the cell suspension was combined with 100 μL of 3 mg/mL Cultrex Rat Collagen I and 12 μL of filtered 1 M NaOH for each gel. Subsequently, 250 μL of the cell-populated collagen gel was transferred to each well of a 24-well dish and incubated at room temperature for 20 min to induce collagen polymerization. CM was added to each well, and the polymerized collagen gel was gently detached from the plate edges. After 24 h, the media was changed to CM, OM, or CaP. Each condition was analyzed in triplicates. Images of the collagen gels were obtained after 5 days. The average contraction values were analyzed by area measurement with imageJ expressed as % reduction in gel diameter compared to the gel diameters without cells.

### (Immuno) fluorescence imaging

Cells were washed with PBS, fixed in 4% PFA for 15 min, and permeabilized for 10 min in 0.5% [v/v] Triton X-100 (Sigma Aldrich). After blocking in 1% bovine serum albumin (BSA), fixed and permeabilized SMCs were incubated with anti-human alpha-smooth muscle actin (α-SMA; 1:200, Dako, M0851), TOM20 (1:200, Proteintech, 11802-1-AP), alpha-tubulin (α-tubulin, 1:25, Cell Signaling, 2144S), calponin (1:50, Thermo Scientific, MA5-32061), mouse IgG control (DAKO, X0931), or rabbit IgG control (R&D systems, AB-105-C). Subsequently, after washing Alexa Fluor 594 (1:1000, Thermo Scientific, R37115) or 488 (1:1000, Life Technologies, A32723) labeled secondary antibody was applied. Nuclei were counterstained with 2.5 μg/ml 4′,6-Diamidino-2-phenylindole dihydrochloride (DAPI, Carl Roth), and slides were covered using a mounting medium (Dako).

For mitochondrial visualization, cells were labeled with 300 nM MitoTrackerRed FM (ThermoFisher Scientific) in serum-free CM at 37°C for 30 min. Nuclear staining was performed with 1 μM Hoechst 33,342 solution (Thermo Scientific). Images were acquired using a Leica DMI6000B inverted fluorescence microscope.

### Real-time extracellular flux analysis

Mitochondrial respiration of cells was characterized by Seahorse XFe96 Flux Analyzer (Agilent) using the Seahorse XF Mito Stress Test Kit (Agilient). This technique allows real-time measurements of the oxygen consumption rate (OCR) and glycolysis (ECAR) in living cells. Cells were seeded into XF96 cell culture microplates (Agilent) at ∼5,000 cells per well. Cells were then cultured for 7 days in CM, OM, or CaP with media change at day 3. One day before the assay, XFe96 Sensor Cartridge (Agilent) was hydrated with water overnight at 37°C in a CO_2_-free incubator. Cells were washed with DMEM supplemented with 10 mM glucose, 2 mM L-glutamine, and 1 mM pyruvate, pH 7.4, and incubated in a CO_2_-free incubator at 37°C for 1 h. The Sensor Cartridge was loaded with different inhibitors from the Seahorse Mito Stress Kit to block the respiratory chain [oligomycin (1 μM), carbonyl cyanide-4 (trifluoromethoxy) phenylhydrazone (FCCP, 0.5 μM), and a mixture of rotenone/antimycin A (0.5 μM)]. The general protocol of the measurements includes three baseline measurements with mix (3 min)/measure (3 min) followed by the injection of port A (oligomycin). Afterward, respiration was measured three times with mix (3 min)/measure (3 min) with the injection of ports B (FCCP) and C (rotenone/antimycin) with the same measurement cycle of three times mix/measure.

The activity of respiratory chain complexes in mitochondria was analyzed in permeabilized cells by Seahorse XFe96 Flux Analyzer. The assay allows direct measurement and overview of respiratory chain activity by adding different substrates to the complexes (14). The measurement was performed as previously described (15). Briefly, cells were washed once with mannitol and sucrose (MAS) buffer [220 mM mannitol, 70 mM sucrose, 10 mM KH_2_PO_4_, 5 mM MgCl_2_, 2 mM N-(2-Hydroxyethyl)piperazine-N’-(2-ethanesulfonic acid) (HEPES) and 1 mM Ethylene Glycol Tetraacetic Acid]. Afterward, MAS buffer supplemented with 4 mM adenosine 5′-diphosphate sodium salt (ADP; Sigma-Aldrich) and 10 μg/ml saponin (Sigma-Aldrich) was added to the cells in a final volume of 180 μl/well. The activity of respiratory chain complexes I, II, and IV was analyzed by adding substrates sequentially during the measurement. To analyze complex I, 10 mM pyruvate (Sigma-Aldrich) and 1 mM malate (Sigma-Aldrich) were directly added to MAS buffer containing ADP and saponin. To inhibit complex I, 20 μM rotenone (Sigma-Aldrich; final concentration 2 μM) was injected *via* port A of XFe96 sensor cartridge. Afterward, 100 mM succinate (Sigma-Aldrich; final concentration 10 mM) for complex II was added *via* port B. Port C and D were loaded with 20 μM antimycin A (Sigma-Aldrich; final concentration 2 μM; port C) as complex II inhibitor and with 1 mM N,N,N’,N’-Tetrametyhl-p-phenylenediamine (TMPD, final concentration 0,1 mM, Sigma-Aldrich; port D) together with 100 mM ascorbic acid (Sigma-Aldrich; final concentrations: 10 mM; port D) to analyze the activity of complex IV. The measurement protocol includes no equilibration step and cycles of two times mix (0.5 min)/wait (0.5 min)/measure (2 min) between injections.

### Phospho kinase array

Tyrosine kinase (PTK) and Serine-Threonine kinase (STK) profiles were determined using the PamChip^®^ peptide tyrosine kinase and Ser/Thr Kinase assay microarray systems on PamStation^®^12, respectively (PamGene International). Each PTK-PamChip^®^ and STK-PamChip^®^ array contains 196 and 144 individual phospho-site(s). Serum-starved (0.1% FBS, 16 h) iSMCs were cultured for 24 h in CM, OM, or CaP (0.1% FBS), washed once in ice-cold PBS, and lysed for 15 min on ice using M-PER Mammalian Extraction Buffer containing Halt Phosphatase Inhibitor and EDTA-free Halt Protease Inhibitor Cocktail (1:100 each; Thermo Scientific). Lysates were centrifuged for 15 min at 16,000 × *g* at 4°C. Protein quantification was performed with Pierce™ Coomassie Plus (Bradford) Assay according to the manufacturer’s instructions.

Pamgene International B.V. supplied all reagents used for PTK and STK activity profiling. For the PTK assay, 7.0 μg of protein was applied per array and assayed using the standard protocol supplied by Pamgene. Initially, to prepare the PTK Basic Mix, the freshly frozen lysate was added to 4 μL of 10 x protein PTK reaction buffer (PK), 0.4 μL of 100 x (BSA), 0.4 μL of 1 M dithiothreitol (DTT) solution, 4 μL of 10 x PTK additive, 4 μL of 4 mM ATP and 0.6 μL of monoclonal anti-phosphotyrosine FITC-conjugated detection antibody (clone PY20). The total volume of the PTK Basic Mix was adjusted to 40 μL by adding distilled water. Before loading the PTK Basic Mix on the array, a blocking step was performed, applying 30 μL of 2% BSA to the middle of every array and washing with PTK solution for PamChip^®^ preprocessing. Next, 40 μL of PTK Basic Mix was applied to each array of the PamChips^®^. Then, the microarray assays were run for 94 cycles. An image was recorded by a CCD camera PamStation^®^12 at kinetic read cycles 32–93 at 10, 50, and 200 ms and end-level read cycle at 10, 20, 50, 100, and 200 ms.

For the STK assay, 1.0 μg of protein and 400 μM ATP were applied per array with an antibody mix to detect the phosphorylated Ser/Thr. The spot intensity at each time point was quantified (and corrected for local background) using the BioNavigator software version 6.3 (PamGene International). Upstream Kinase Analysis, a functional scoring method (PamGene), was used to rank kinases based on combined specificity scores (based on peptides linked to a kinase, derived from six databases) and sensitivity scores (based on treatment-control differences) (16).

### *In silico* drug repurposing analysis

The common genes between OM and CaP-calcified SMCs from the transcriptomics datasets were used as the gene expression signature for drug repurposing analysis using the web-based tool Connectivity Map (CMap^2^) (17). The two different lists of up and down-regulated genes were submitted in the “Query” of CMap tool against the Touchstone reference dataset of gene expression (L1000) to compare the query gene set with compounds reference perturbagen signatures (January 2021). The compounds were ranked according to the CMap score (tau score). A negative score indicated that the compound had a potentially reversed gene signature profile. A positive score indicated that the compound potentially mimics the input gene-phenotype. Compounds with a tau score < or > 70 and a *p*-value < 0.05 were considered for further investigation. The sum of the compound tau score in OM and CaP signatures was used to rank the compounds.

### Human carotid artery specimens and transcriptomic analysis

The human biocollection and transcriptomic analysis have been published previously (18). From February 2008 to December 2015, atheromatous plaques were harvested and collected from patients undergoing carotid endarterectomy in the Department of Vascular Surgery at Nantes University Hospital. Healthy arteries free of atherosclerotic lesions were obtained from organ donors. Sample collection and handling were performed under the Medical and Ethical Committee guidelines in Nantes, France, and written informed consent was obtained from all patients and organ donors. The experimental protocol was approved by the Agence de Biomédecine (research protocol #PFS09–014, authorized on Dec 23, 2009, by the Agence de Biomédecine, France). Legal and ethical authorizations were granted by the French Research Ministry (n° DC-2008–402), the National Commission for Computerized Information and Liberties (CNIL, n° 1520735 v 0), and the local ethical committee (GNEDS).

The atherosclerotic plaques were fixed in 10% formalin for 24–48 h, decalcified in Sakura TDE 30 fluid, and embedded in paraffin. Sections (4 μm thickness) were stained with hematoxylin-eosin (HE). Whole sections were imaged with a NanoZoomer digital slide scanner (Hamamatsu Photonics, Hamamatsu, Japan).

Samples for RNA processing were harvested and immediately snap-frozen in liquid nitrogen or stored in All-protect Tissue Reagent (Qiagen). Total RNA was extracted using Macherey Nagel NucleoSpin columns (Macherey Nagel). RNA was hybridized to Agilent Human Gene Expression Microarrays. Fluorescence values corresponding to raw expression data were extracted using Feature Extraction Software (Agilent). Positive and negative control probes were removed. Non-linear effects, such as background or saturation, were corrected by Lowess against a median profile of all samples. Values of replicate probes were averaged. Genes differentially expressed between atherosclerotic and healthy arteries were identified using Significance Analysis of Microarrays, with an FDR = 0% (19). Microarray data have been deposited in NCBI’s Gene Expression Omnibus (GEO) and are accessible through GEO Series accession number GSE100927.

### Human coronary artery plaque single-cell ribonucleic acid sequencing data analysis

Single-cell RNA sequencing data from human coronary artery plaques were previously published (20) (GEO Series accession number GSE131778) and plotted using the web-based tool PlaqView^3^ (21). In brief, diseased specimens from the right coronary artery with atherosclerotic lesions ranging from mild, non-calcified plaques to more advanced lesions with areas of calcification of four cardiac transplant recipients were dissociated and subjected to single-cell RNA sequencing (20). The clinical characteristics of the patients included in the study were previously described and included written consent prior to the procedure (20).

### Mouse artery specimens and transcriptomic analysis

Data used in this study were previously published (22) and are available in NCBI’s GEO (accession number GSE159833). We used publically available RNA sequence data of the artery of Apoe-deficient mice as a model for atherosclerotic intimal calcification and a 5/6 nephrectomy-induced CKD model for medial calcification. Briefly, *Apoe*-deficient mice were fed a chow diet for 16 months to induce atherosclerotic intimal calcifications. 8-week-old C57BL/6 mice were subjected to subtotal 5/6 nephrectomy using a two-step method and fed a diet containing 2% phosphate and water containing 0.45% NaCl for 4 weeks after 5/6 nephrectomy to induce medial calcification. 8-week-old C57BL/6 mice fed a chow diet were used as control. RNA sequencing data were available for two samples per group. Data were extracted and differentially regulated genes (Log2 fold change < −0.5 or > 0.5; *p* < 0.05) in *Apoe*-deficient mice or CKD group compared to the control group were identified using the web-application GREIN (GEO RNA-seq Experiments Interactive Navigator^4^) (23).

### Statistical analysis

Statistical analyses were performed using the GraphPad Prism program (Prism Software Inc., Version 8). Data are presented as mean ± SD; *n* indicates the number of independent experiments. For comparison between two groups, unpaired Student’s *t*-test was performed. For comparison among three or more treatment groups, one-way ANOVA followed by Dunnett posttest was performed. In case of unequal variance detected by *F*-test, unpaired Student’s *t*-test with Welch’s correction was used. A *p*-value of less than 0.05 was considered significant.

## Results

### Characterization of osteogenic medium and calcium phosphate-induced calcification

**FIGURE 1:**
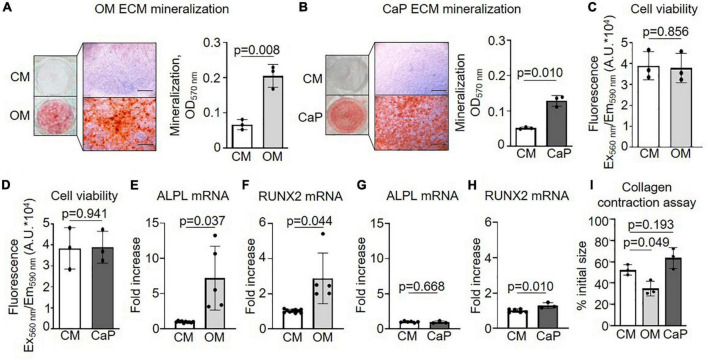
Characterization of osteogenic medium (OM) and calcium phosphate (CaP)-induced calcification of primary coronary artery smooth muscle cells (pSMCs). **(A)** pSMCs were cultured in control medium (CM) and OM for 21 days or **(B)** CaP for 7 days. Representative images of extracellular matrix (ECM) mineralization were detected by alizarin red S staining and eluted staining quantified by assessing the optical density at 570 nm. Scale bar: 1,000 μm. *n* = 3 **(C)** Effect of OM and **(D)** CaP-induced calcification on cell viability assessed by AlamarBlue assay (Fluorescence Ex_560_ nm/Em_590_ nm) on day 21 for OM and day 7 for CaP. *n* = 3. **(E–H)** ALPL and RUNX2 mRNA expression for **(E,F)** OM-calcified pSMCs (day 7) and **(G,H)** for CaP-calcified pSMCs (day 3). **(I)** Collagen contraction assay at day 5. *n* = 3–5. Error bars indicate ± SD. Each n indicates an independent pSMC donor. Unpaired *t*-test and one-way ANOVA with Dunnett’s *post hoc* test for **I**.

First, we examined the calcification profile of primary smooth muscle cells (pSMCs) cultured with osteogenic media (OM) or calcium phosphate media (CaP). Calcification occurred in both media yet differed concerning amount and kinetics. Alizarin red S staining and quantification showed that calcification was 3.0 fold increased at day 21 in OM (*p* = 0.008) and 2.5 fold increased at day 7 in CaP (*p* = 0.010) (Figures 1A,B). Calcification was first observed after 14 days of culture in OM and after 5 days in CaP, respectively (Supplementary Figures 1A,B). OM and CaP did not affect cell viability at day 14 and 7, respectively (Figures 1C,D). Next, we assessed mRNA levels of the osteogenic markers ALPL and RUNX2 at early time points. ALPL mRNA (7.2 fold, *p* = 0.037) and RUNX2 mRNA (2.8 fold, *p* = 0.044) was up-regulated in OM at day 7, while in CaP-calcified SMCs, we observed a 1.3 fold increase of RUNX2 mRNA (*p* = 0.010) and no change in ALPL mRNA at day 3 (Figures 1E–H). Tissue alkaline phosphatase (TNAP) activity increased 3.7-fold (*p* = 0.010) in OM but not in CaP on day 14 (Supplementary Figure 1C). We performed a collagen gel contraction assay to compare the effects of OM and CaP in SMC contractility. When placed into a collagen gel, SMCs exert traction forces to remodel their local environment, reducing the gel area by consolidating collagen fibrils (24). Control SMCs contracted the gels by 52% of the initial area over 5 days (Figure 1I). OM significantly decreased the % of the initial collagen gel size compared to CM (−1.4 fold, *p* = 0.049), while CaP tended to increase the gel size (1.2 fold, *p* = 0.193).

### Osteogenic medium and calcium phosphate-calcifying primary vascular smooth muscle cells display distinct gene expression

**FIGURE 2:**
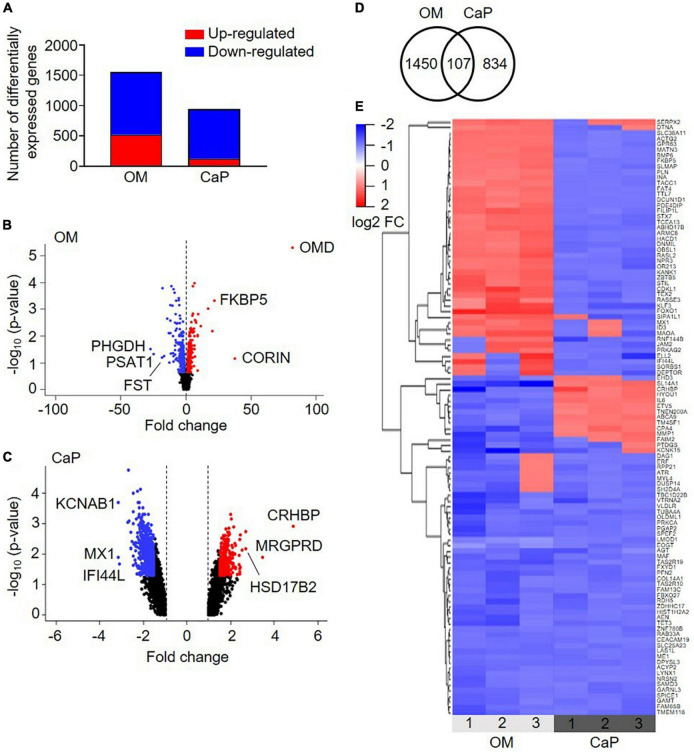
Transcriptional profiling of osteogenic (OM) and calcium phosphate (CaP)-calcified primary coronary artery smooth muscle cells (pSMCs). **(A)** Number of up-regulated (red) and down-regulated (blue) genes from OM and CaP-calcified pSMCs compared to control (CM). Cut-off FC 1.5. **(B,C)** Volcano plots of the differentially expressed genes in OM and CaP-calcified pSMCs, respectively, compared to CM. Plotted on the *x*-axis is the fold change between calcified and CM pSMCs. Plotted on the *y*-axis is the –log_10_(*p*-value). Significant differentially expressed genes are divided into up-regulated (red dots) and down-regulated (blue dots) genes, while non-significant genes are shown in black. The top three up-and downregulated genes are named. **(D)** The Venn diagram shows overlapping differentially expressed genes between OM and CaP-calcified pSMCs. **(E)** Heatmap presenting the expression profiles of the 107 common genes. Genes with a fold change ≥ ± 1.5 and a *p*-value < 0.05 are considered differentially expressed. *n* = 3 independent primary SMC donors.

To study the underlying molecular mechanisms driving the different VC models, we analyzed the transcriptome of CaP and OM-calcified pSMCs on days 3 and 7, respectively, aiming to achieve comparable early calcification time points corresponding to one-third of the time required to achieve calcification with the respective protocols (Supplementary Figure 1). Applying a fold-change cut-off of 1.5 identified 1,557 differentially regulated genes in OM-calcified pSMCs and 941 genes in CaP-calcified pSMCs compared to control media (CM) (Figures 2A–C and Supplementary Table 2).

Pathway over-representation analysis of the OM-regulated genes highlighted the enrichment of elastic fiber formation, elastic fiber structural molecules, and metabolism pathways (Supplementary Table 3). In CaP, the top over-represented pathways were phosphatidylinositol signaling system, regulation of pyruvate dehydrogenase complex, and SMC contraction (Supplementary Table 4).

We combined the transcriptomics datasets of the differentially regulated genes from OM and CaP-calcified pSMCs and found 107 genes shared between the two data sets (Figure 2D). 1/3 of the genes showed similar gene expression, while 2/3 exhibited a distinct gene regulation cluster (Figure 2E). Pathways associated with the over-represented genes included muscle contraction, SMC contraction, and epithelial growth factor receptor (EGFR) transactivation by gastrin (Supplementary Table 5). These data suggested that OM and CaP-calcified pSMCs displayed distinct gene profiles driving calcification along distinct pathways.

### Establishment and characterization of an immortalized vascular smooth muscle cell model

We generated an immortalized SMC line (iSMC) to avoid the intrinsic biological variability associated with primary SMCs. Validation of the iSMC phenotype revealed expression levels of the contraction marker calponin and SMC markers α-SMA and α-tubulin similar to pSMCs (Supplementary Figure 2). Characterization of OM and CaP-calcified iSMCs related to TNAP activity (Supplementary Figure 3A), ECM mineralization (Supplementary Figures 3B,C), mRNA expression of the osteogenic markers ALPL and RUNX2 (Supplementary Figures 3D–F), cell viability (Supplementary Figures 3H–J), and contraction properties (Supplementary Figure 3K) showed similar results in iSMCs compared to primary SMCs.

### Osteogenic medium and calcium phosphate-calcifying vascular smooth muscle cells display a different mitochondrial function profile

**FIGURE 3:**
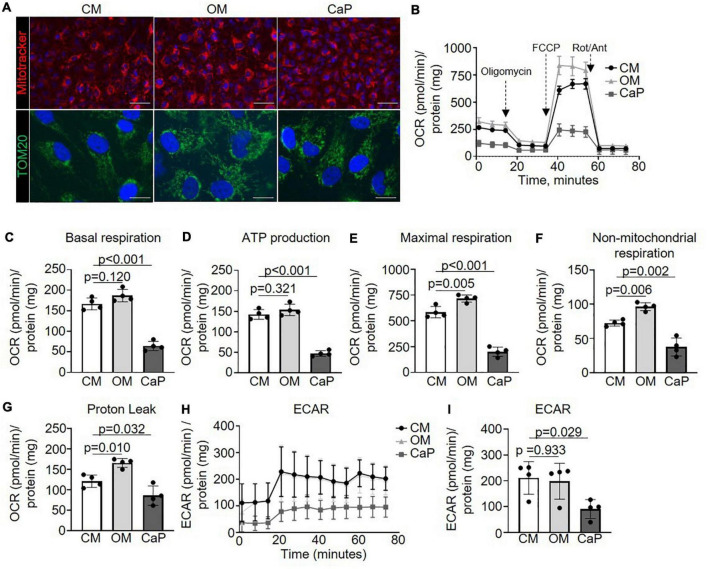
Mitochondrial respiration increases in osteogenic medium (OM) and attenuates in calcium phosphate (CaP)-calcified vascular smooth muscle cells (SMC) Immortalized SMCs (iSMCs) were cultured for 7 days in control medium (CM), OM or CaP. **(A)** Representative images of live iSMCs for the mitochondria-specific dye MitoTracker Red (red) and nuclear Hoeschst staining (blue) and immunofluorescence for TOM20 (green) with DAPI nuclear staining (blue). *n* = 3, scale bar: 75 μm and 10 μm, respectively. **(B)** Mitochondrial oxygen consumption rates (OCR) of OM and CaP-calcified iSMCs subjected to the XF Mito Stress Test measured using the Seahorse XF96 flux analyzer, with sequential injections of mitochondrial effectors [oligomycin, carbonyl cyanide-4 (trifluoromethoxy) phenylhydrazone (FCCP) and rotenone (Rot), antimycin (Ant)] at time points indicated by the downward arrows. *n* = 4. **(C)** Basal respiration, **(D)** ATP production. **(E)** Maximal respiration. **(F)** Non-mitochondrial respiration. **(G)** Proton leak. **(H,I)** Extracellular acidification rate (ECAR) and quantification. OCR and ECAR were normalized to protein content. Error bars indicate ± SD. Each n indicates an independent replicate. One-way ANOVA with Dunnet’s *post hoc* test.

Metabolism and pyruvate dehydrogenase complexes were two pathways highlighted in our over-representation analysis. Both pathways inform about mitochondrial function and, thus about, cell health (25). Mitochondria are critically required in energy-demanding functions, and the mitochondrial matrix is rich in calcium (26), which has been shown to trigger calcification (27). Therefore, we investigated the effect of OM or CaP on the morphology and bioenergetics of mitochondria. MitoTracker and TOM20 staining showed no difference in the mitochondria phenotype in calcifying iSMCs (Figure 3A). However, we observed different alterations in mitochondrial function for OM and CaP-calcifying iSMCs using real-time extracellular flux analysis to evaluate the mitochondrial respiration and glycolysis by simultaneous time-course measurement of the oxygen consumption rate (OCR) and extracellular acidification rate (ECAR), respectively. Compared to CM, CaP decreased the basal respiration (−62%, *p* < 0.001), the ATP production (−67%, *p* < 0.001), maximum respiration (−66%, *p* < 0.001), non-mitochondrial respiration (−49%, *p* = 0.002), and proton leakage (−28%, *p* = 0.032) in iSMCs (Figures 3B–G). On the other hand, OM increased maximal respiration (+ 22%, *p* = 0.005), non-mitochondrial respiration (+ 32%, *p* = 0.006), and proton leakage (+ 36%, *p* = 0.010) compared to CM. Glycolysis was 2.3-fold decreased in CaP-calcified iSMCs compared to CM (*p* = 0.029), while OM did not alter glycolysis (Figures 3H,I).

To further explore the mechanisms associated with the metabolic impact of OM and CaP, we evaluated single mitochondrial complex function (Supplementary Figure 4A). Through mitochondrial efflux analysis, we observed that OM-calcified iSMCs displayed increased complex I OCR (+ 39%, *p* = 0.025), and IV OCR (+ 47%, *p* = 0.011) OCR, while CaP-calcified iSMCs showed a tendency to reduced complex III (−29%, *p* = 0.071) and IV (−32%, *p* = 0.068) (Supplementary Figures 4B–E).

### Drug repurposing

**FIGURE 4:**
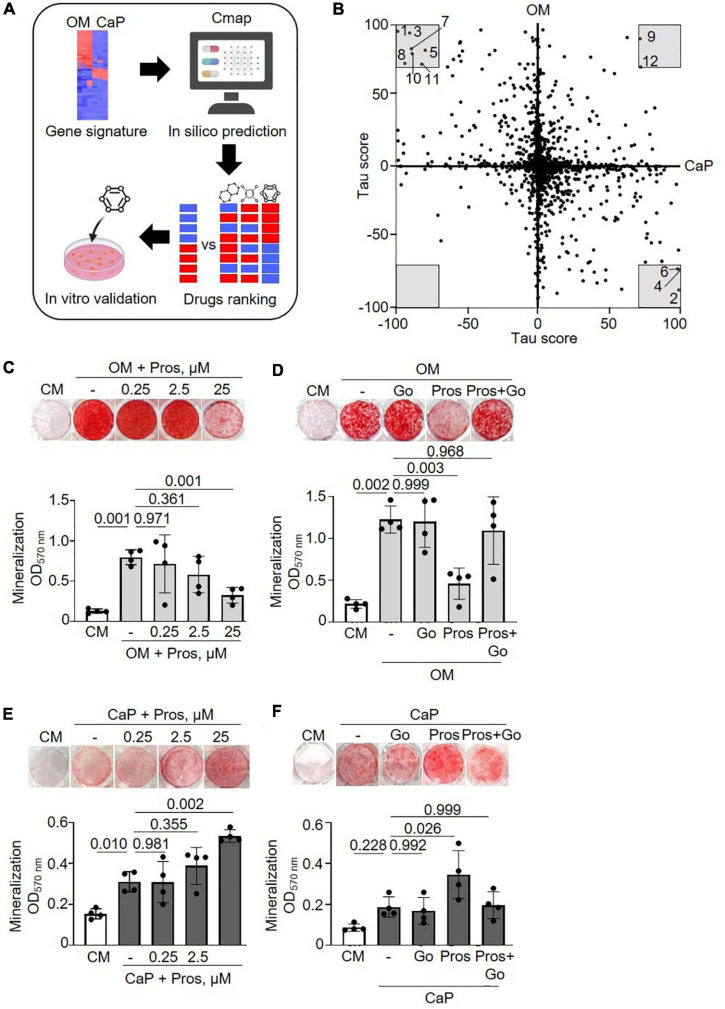
Drug repurposing of osteogenic medium (OM) and calcium phosphate (CaP) gene signatures. **(A)** Scheme of the method. Created with https://Biorender.com **(B)** Scatter plot with predicted effects of drugs on OM and CaP-induced calcification. Gray area–drugs with predicted tau score < or > 70. 1: PD-184352, 2: Prostratin, 3: Fluticasone, 4: Ingenol, 5: L-690330, 6: Phorbol 12-myristate 13-acetate (PMA), 7: Halometasone, 8: Sunitinib, 9: MLN-2238, 10:Hydrocortisone, 11: Benzatropine, 12: Puromycin. **(C,D)** Effect of the protein kinase C (PKC) activator prostratin (Pros) and the PKC inhibitor Go6983 (Go) in OM and **(E,F)** in CaP-calcified immortalized vascular smooth muscle cells (iSMCs). iSMCs were cultured in control medium (CM) and OM for 14 days or CaP for 7 days with different concentrations of prostratin (0.25, 2.5, and 25 μM). Go (100 nM) was combined with 25 μM prostratin. Representative images of extracellular matrix mineralization (top) detected by alizarin red S staining and quantification of eluted staining (bottom). *n* = 4–5. C-H, DMSO (1:1,000) was used as solvent control in CM, OM, and CaP groups. Error bars indicate ± SD. Each *n* indicates an independent replicate. One-way ANOVA with Dunnett’s *post hoc* test.

We performed a drug repurposing analysis using the Cmap database to identify novel compounds targeting VC and to verify the hypothesis that they would work differently in our *in vitro* models. Cmap analysis matches a specific disease’s transcriptomic profiles (differentially induced and repressed genes) with a signature profile associated with compounds. We independently scored the common gene profiles of OM and CaP datasets (Figure 4A). When a compound signature reversely correlates with the differentially expressed genes, the compound is considered a potential repositioning candidate. If it correlates positively, it is considered a molecular mimic of the observed phenotype (17).

Consequently, we focused on the compounds with the highest connectivity and retained those with a tau score > 70 or < –70 in both OM and CaP datasets, which resulted in 12 overlapping candidate compounds (Figure 4B and Table 1). Interestingly, no compound displayed simultaneous negative connectivity for OM and CaP. Two out of 12 compounds exhibited positive connectivity for OM and CaP-calcified iSMCs. Ten out of 12 compounds displayed inverse connectivity (three compounds: negative connectivity for OM, positive connectivity for CaP; seven compounds: positive connectivity for OM, negative connectivity for CaP) (Table 1), highlighting the distinct regulation among the same genes.

**TABLE 1:** Repurposable drug candidates with tau score < or > 70 sorted by the sum of tau scores in osteogenic media (OM) and calcium phosphate media (CaP) gene signature (tau distance).

No.	Drug	OM	CaP	Tau distance	Function
1	PD-184352	95.3	−98.8	194.1	MEK inhibitor
2	Prostratin	−87.2	99.5	186.7	PKC activator
3	Fluticasone	94.1	−90.3	184.5	Glucocorticoid receptor agonist
4	Ingenol	−73.0	99.7	172.7	PKC activator
5	L-690330	82.9	−89.0	172.0	Inositol monophosphatase inhibitor
6	PMA	−72.4	98.8	171.2	PKC activator
7	Halometasone	79.5	−88.6	168.1	Glucocorticoid receptor agonist
8	Sunitinib	72.6	−93.8	166.4	FLT3 inhibitor
9	MLN-2238	90.0	72.3	162.3	Proteasome inhibitor
10	Hydrocortisone	81.9	−79.4	161.4	Glucocorticoid receptor agonist
11	Benzatropine	72.1	−81.8	153.9	Acetylcholine receptor antagonist
12	Puromycin	72.6	70.2	142.8	Protein synthesis inhibitor

Next, we calculated the distance of tau scores from OM and CaP and selected the top five compounds for further *in vitro* validation. Among the top five compounds were two protein kinase C (PKC) activators –prostratin and ingenol –which are predicted to promote calcification in CaP and inhibit calcification in OM. In line with the findings, results from the phospho-kinase array analysis from OM and CaP-treated iSMCs also indicated a differential role of the PKC signaling pathway in the calcification process (Supplementary Figures 5A–C). OM and CaP-treated SMCs shared nine differentially regulated serine/threonine kinases and 64 differentially regulated tyrosine kinases (Supplementary Figure 5A).

Subsequently, we performed *in vitro* experiments to validate the role of PKC. Both prostratin and ingenol decreased ECM mineralization in OM (−2.9 fold, *p* = 0.001 and −5.6 fold, *p* = 0.005, respectively) (Figure 4C and Supplementary Figure 6A), which was abolished by the PKC inhibitor Go6983 (Figure 4D and Supplementary Figure 6B), supporting the predicted effect from the *in silico* analysis. In CaP-calcified iSMCs prostratin and ingenol increased ECM mineralization (+ 1.8 fold, *p* = 0.002 and + 1,5 fold, *p* = 0.034) (Figure 4E and Supplementary Figure 6C) which was abolished by the PKC inhibitor Go6983 (Figure 4F and Supplementary Figure 6D).

Regarding the other repurposable compound candidates, the MEK inhibitor PD-184352 displayed no effect on ECM matrix mineralization of both OM and CaP models (Supplementary Figures 7A,B). Using the glucocorticoid receptor agonist fluticasone, which was predicted to increase OM and decrease CaP-induced SMC phenotype, we observed increased ECM mineralization when used as a dexamethasone substitute in OM and no effect on CaP-calcified iSMCs (Supplementary Figures 7C,D). Finally, L-690330, an inositol monophosphatase inhibitor, prevented ECM mineralization in both OM and CaP-treated cells (Supplementary Figures 7E,F). In summary, we validated two out of five candidates (Supplementary Figure 7G). The tested compounds did not interfere with cell viability (Supplementary Figures 8A–L).

### Transcriptome-wide comparison of the gene signature of *in vitro* calcification models with mouse and human calcified arteries

Finally, we compared our *in vitro* OM and CaP gene signature with the gene signature of mouse and human calcified arteries to evaluate the relevance of each model and the common genes involved in both *in vitro* and *in vivo* calcification.

**FIGURE 5:**
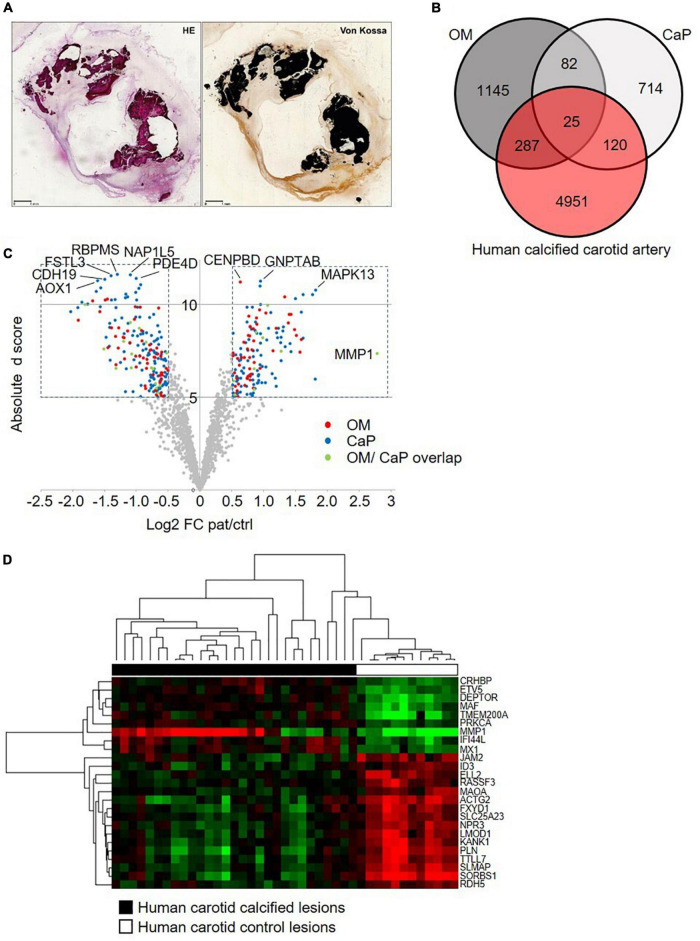
Comparative transcriptome analysis across human carotid artery and *in vitro* models. **(A)** Serial sections of a resin embedded carotid lesion [hematoxylin eosin staining (HE) and von Kossa staining] Bar: 1 mm. **(B)** Venn diagram comparing the osteogenic media (OM)-treated primary coronary artery smooth muscle cells (pSMCs) and calcium phosphate (CaP)-treated pSMCs gene signatures to the human calcified carotid artery gene signature. **(C)** The volcano plot of the human calcified carotid artery dataset displays only the OM and CaP gene signature genes. Plotted on the *x*-axis is the log_2_ of the fold change (FC) between the calcified and control carotid artery. Plotted on the *y*-axis is the absolute *d* score obtained through Significant Analysis of Microarrays (*T*-statistic value). Significant differentially expressed genes (Absolute d score > 5, Log_2_ FC < –0.5 or > 0.5) are indicated in red (OM genes), blue (CaP genes), or green (shared between OM and CaP). Non-differentially expressed genes are shown in gray. **(D)** Hierarchical clustering of the 25 differential regulated genes that are common between carotid artery, OM-calcified pSMCs, and CaP-calcified pSMCs. Green, black and red corresponds to lower, median and higher gene expression values, respectively.

We used human carotid atherosclerotic lesions from the ECLAGEN biocollection that displayed calcification as detected by histology (Figure 5A; 18). Transcriptome analysis of all sets of genes showed a panel of 25 common genes that were differentially expressed in both *in vitro* models and diseased carotid arteries (Figures 5B,C and Supplementary Tables 6, 7). Nine common genes were upregulated in calcified carotid lesions and 16 down-regulated (Figure 5D). DAVID analysis identified four main enriched gene ontology (GO) clusters, including GO terms like muscle contraction, cytoskeleton, cytoplasm, mitochondria, membrane, protein binding, nucleotide-binding, and transcription regulation (28). Furthermore, an additional 287 and 120 regulated genes were shared between human lesions and OM and CaP models, respectively, suggesting that both models share molecular features related to plaque calcification.

Furthermore, we used publically available single-cell RNA sequencing data from the human coronary artery to localize the 107 common genes between OM and CaP-calcified pSMCs to specific cell clusters. 102 from the 107 genes were present in the 9,798 cells that were previously annotated to 14 cell clusters (20; Supplementary Figure 9). Considering only genes that were expressed in at least 20% of the cells revealed 53 genes whose highest expression was detected in the SMC, pericyte 1, fibromyocyte, and fibroblast cell clusters (Supplementary Table 8).

Next, we used publically available transcriptome data from an *Apoe*^–/–^ and CKD mouse model to address intimal and medial calcification. Intersecting differentially regulated genes from OM and CaP-calcified pSMCs and *Apoe*^–/–^ and CKD mice revealed 26 genes shared between the four data sets (Supplementary Figure 10A). Considering differentially regulated genes, OM shared 42.7% of its genes with *Apoe*^–/–^ mice and 23.0% with CKD mice. CaP shares 39.7% of its genes with Apoe-deficient mice and 20.4% with CKD mice (Supplementary Figure 10B).

## Discussion

Vascular calcification comprises mineral deposition in the tunica media or the tunica intima, associated with distinct risk factors and clinical outcomes (29). Intimal calcification generally correlates with atherosclerosis plaque burden, hyperlipidemia, and chronic arterial inflammation. In contrast, medial calcification is a non-occlusive process that leads to increased vascular stiffness and reduced vascular compliance, frequently associated with diabetes and chronic kidney disease (3). To our knowledge, this work compares for the first time different *in vitro* SMC calcification protocols mimicking intimal and medial calcification, respectively. By deploying different *in vitro* models, our results support the hypothesis that different molecular pathways trigger intimal and medial calcification.

It is a widely accepted concept that the development of VC is associated with phenotypic transdifferentiation of SMCs, resulting in SMCs with osteoblast-like characteristics (30). Our data corroborate published findings, demonstrating that TNAP activity and ALPL mRNA levels are induced in OM-calcified SMCs (31). Interestingly, TNAP was not regulated in CaP-calcified SMCs, consistent with previous reports demonstrating repression of TNAP activity and mRNA expression in CaP-calcified SMCs at early calcification time points (32). Moreover, TNAP was not present in extracellular vesicles isolated from CaP-calcified SMCs (33). This suggests that CaP-induced calcification is independent of TNAP-mediated osteogenesis.

Changes in the transcriptional profile of SMCs have been reported during osteogenic differentiation (34, 35). Our gene expression analysis revealed distinct molecular regulation in mRNA profiles in response to OM or CaP-induced calcification. OM and CaP share less than 12% of their differentially regulated genes. Those shared genes between OM and CaP-calcified SMCs highlighted enrichment of genes encoding proteins for smooth muscle contraction and were mostly oppositely regulated. Based on a functional collagen contraction assay, we observed that SMC contraction dynamics were oppositely affected by OM and CaP-induced calcification, where OM displayed higher contraction rates than control. One of the primary functions of SMCs is maintaining vascular tone and regulating blood pressure *via* their contractile properties (36). Upon biological stress signals or vascular injury, SMCs undergo a phenotypic modulation associated with higher proliferation rates, migration, and altered contractile marker expression (37).

Our transcriptomics pathway enrichment analysis revealed alterations in the metabolism pathway in OM-calcified SMCs and changes in the pyruvate dehydrogenase complex in CaP-calcified SMCs. The pyruvate dehydrogenase complex is central in regulating energy metabolism and mitochondrial function (38). Furthermore, mitochondrial dysfunction has been associated with VC progression (39). Therefore, we investigated whether OM and CaP-induced SMC calcification was related to alterations in mitochondrial bioenergetic properties. We observed that CaP-calcified SMCs showed apparent impairment of mitochondria phosphorylation parameters, raising the question of whether high concentrations of CaP directly attenuate mitochondrial function. Calcium is considered an important regulator of mitochondrial metabolism, and isolated mitochondria increase ATP production upon stimulation with low calcium levels (40). Conversely, higher concentrations of CaP result in mitochondrial calcium overload and attenuated oxidative phosphorylation. Recent studies attribute this adverse effect to intramitochondrial calcium phosphate granules (41, 42). In calcified SMCs a shift from mitochondria phosphorylation toward a glycolytic breakdown of glucose was previously described (43). This change is similar to the Warburg effect, frequently observed in cancer cells. Notably, the reduction of mitochondrial respiration in CaP-calcified SMCs was also accompanied by a decrease in glycolysis, which was not observed in OM-calcified SMCs. In a murine model of phosphate-induced VC, others also demonstrated that decreased mitochondrial phosphorylation precedes decreased glycolytic capacity (44). This suggests that in CaP-calcified SMCs, glycolysis might not compensate for deficient mitochondrial ATP production.

Furthermore, we found that treating SMC with OM enhanced maximal respiratory rate. Others have shown that the transdifferentiation of mesenchymal stem cells into osteoblasts is also linked to increased mitochondrial respiration and that human aortic SMCs displayed elevated basal respiration after ß-glycerolphosphate treatment, a component of OM (26, 45). Therefore, the increased oxidative phosphorylation in OM-calcified SMCs potentially highlights a mitochondrial response to maintain the high energy demands of ECM synthesis, remodeling, and contraction. Nevertheless, the exact mechanisms underlying mitochondrial dysfunction during VC have yet to be elucidated. Current literature suggests mitochondrial dysfunction as both a cause and consequence of VC (46). Previous data demonstrated a diffuse tubulin cytoskeleton and a more apparent actin cytoskeleton in OM-calcified SMCs (47). Interestingly, there is evidence from yeasts that cytoskeletal changes can be transmitted to mitochondria, resulting in functional modification of the organelle (48, 49). Whether the observed cytoskeleton alterations and mitochondrial functional changes are linked in OM or CaP-calcified SMCs remains to be further investigated.

Although significant progress has been made in understanding VC pathology, VC remains a disease without therapy. Drug repurposing may indeed identify novel therapeutic use for existing drugs. This *in silico* approach was applied in cardiovascular disease and COVID-19 (50–52). In this study, the drug repositioning pipeline revealed a differential role of PKC in VC. We validated those results using prostratin and ingenol, specific PKC activators. As predicted, the PKC activators reduced calcification in OM and increased it in CaP *in vitro*. Concomitantly, the PKC signaling was shown in our kinome array as differentially regulated in OM and CaP-calcified SMC, suggesting that although oppositely regulated, the PKC signaling pathway is involved in both intimal and medial calcification.

Indeed, increasing evidence suggests that PKC is potentially involved in the process of CVD. PKCδ expression was induced in human atherosclerotic plaques from the mammary artery compared to control tissue (53). PKCβ deficient mice or treatment with the PKCβ inhibitor ruboxistaurin decreased the atherosclerotic lesion size in Apoe-deficient mice (54). Additionally, inhibition of PKC resulted in a dose-dependent inhibition of dexamethasone-induced osteogenic differentiation and ECM calcium deposit in human mesenchymal stem cells (55). Other studies suggested that PKCα suppresses bone formation (56, 57). This is in line with our results demonstrating that the PKC activators ingenol and prostatin inhibit OM-mediated SMC calcification. Contrary to our results, in human SMCs calcified using 2 mM calcium and 5 mM β-glycerophosphate, deletion or inhibition of PKCα increased ECM mineralization (58). Others also showed that PKCα and δ phosphorylation was decreased in phosphate-induced calcification of SMCs and aortas through osteogenic signaling and cytoskeleton disruption (59). Although our results indicate opposite regulation between PKC and VC in CaP-mediated calcification, it is important to mention that we identified and used compounds to activate all PKC isoforms since prostratin and ingenol have little PKC isoform selectivity.

While *in vitro* models have proven to be important in biological research, it remains challenging to translate the results to human conditions. Mapping the common differentially regulated genes from OM and CaP-calcified SMCs to single-cell RNAseq data from human coronary arteries showed that most of the genes were present in the cell clusters annotated for SMCs, pericytes, fibromyocytes, and fibroblasts. Those clusters also contained the osteogenic markers TNFRSF11B (20), ALPL, and MSX2. Differentially regulated genes from OM and CaP and the gene signature of human calcified carotid arteries shared only 25 genes. The observed transcriptional dissimilarities *in vitro* and *in vivo* are expected since the atherosclerotic plaque consists of multiple interacting cell types with crosstalk to the ECM that may drive different transcriptional changes (60). Using two different vascular beds–coronary artery SMCs and carotid arteries–might further explain the diverse mRNA profiles. Previously, it has been shown that the calcification propensity is highly dependent on the heterogeneity of SMCs from different vascular beds underlying different calcification mechanisms (61).

Our study has limitations. VC is a complex disease caused by many risk factors, such as diabetes, chronic kidney disease, and aging, and multiple cell types and differentiation pathways are involved that our study did not address. Moreover, while differentiating micro and macrocalcification is crucial to atherosclerotic plaque vulnerability (62), the *in vitro* SMC models cannot discriminate between the two calcifications morphologies. Spotty microcalcification within the intimal atherosclerotic plaque promotes plaque vulnerability, while macrocalcification is discussed to stabilize the plaque (63). The sheet-like medial calcifications cause increased vascular stiffness and reduced vessel compliance (62). Those differences in the clinical consequences between medial and intimal calcification underline future efforts to adopt the calcification disease models to study the underlying mechanisms. Furthermore, *in vitro* cell culture models do not recapitulate the interplay of different cell types and the crosstalk with the ECM that is important for developing micro-and macrocalcification. Previously, it was shown that the calcification morphology and density depend on the plaques’ collagen content (64). Previously, our group reported that integrating different omic layers can yield novel molecular pathways that are not visible at a single level in cardiovascular calcification (65). Here, only one single molecular layer at one timepoint was accessed through transcriptomics and further used in the *in silico* drug repurposing analysis. It is well-acknowledged that not one single model can imitate the disease environment (6). The rationale of selecting the most suitable *in vitro* vascular calcification model may allow a precise understanding of the data and elaborate the initial development of novel drugs for the treatment. In the future, *in vivo* validation is needed to support the differential mechanisms of calcification in the tunica intimal and media.

## Conclusion

**FIGURE 6:**
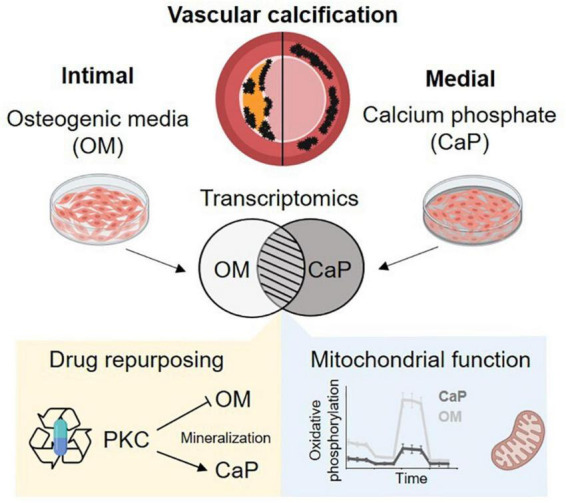
Summary schema. Osteogenic medium (OM) and calcium phosphate medium (CaP) trigger distinct mechanism of calcification. We found differential regulated mitochondrial function and a differential role of protein kinase C (PKC). Created with https://Biorender.com.

Our results strengthen previous observations that OM and CaP-induced SMC calcification *in vitro* is triggered by different mechanisms (Figure 6). We found a differential role of PKC in OM and CaP-calcified SMCs, providing new opportunities for therapeutic investigation. Our data suggest that the field should limit the generalization of results found in *in vitro* studies. Proper method reporting of *in vitro* calcification protocols could lead to a better understanding of the potential mediators of VC and yield significant insights into the pathophysiological mechanism of intimal and medial calcification.

## Data availability statement

The datasets presented in this study can be found in online repositories. The names of the repository/repositories and accession number(s) can be found in the article.

## Ethics statement

The studies involving human participants were reviewed and approved by Nantes University Ethical Committee (GNEDS). The patients/participants provided their written informed consent to participate in this study.

## Author contributions

CG and TQ conceptualized and designed the study. TQ, RK, and NM contributed to the study design and the discussion of the results. MH, AB, JH, EvdV, and MG performed the experiments. MH and MS performed the statistical analysis. BK, DM, YG, and WJ-D contributed to the interpretation and the discussion of the results. MH created the figures and wrote the first draft of the manuscript with assistance from CG, JH, and AB. CG, WJ-D, and TQ revised the manuscript. All authors contributed and reviewed the manuscript and approved the final version.

## Footnotes

1
http://consensuspathdb.org

2
https://clue.io/

3
http://plaqviewv2.uvadcos.io/

4
http://www.ilincs.org/apps/grein/
