# Supplementary material for: Distinct role of mitochondrial function and protein kinase C in intimal and medial calcification in vitro
Source: Front Cardiovasc Med. 2022 Sep 20;9:959457. doi: 10.3389/fcvm.2022.959457 (PMC9530266; doi:10.3389/fcvm.2022.959457)
Supplement: Supplementary file 1 [file Data_Sheet_1.PDF]

Supplementary Material

Distinct role of mitochondrial function and protein kinase C in intimal and medial calcification in vitro

Marina A. Heuschkel[1], Anne Babler[2], Jonas Heyn[1], Emiel P.C. van der Vorst[3-6], Marja Steenman[7], Maren Gesper[1], Ben Kappel[1], David Magne[8], Yann Gouëffic[9], Rafael Kramann[2, 10, 11], Willi Jahnen-Dechent[12], Nikolaus Marx[1], Thibaut Quillard[7,13], Claudia Goettsch[1*]

[1] Department of Internal Medicine I – Cardiology, Medical Faculty, RWTH Aachen University, Aachen, Germany
[2] Institute of Experimental Medicine and Systems Biology, University Hospital, RWTH Aachen, Aachen, Germany
[3] Interdisciplinary Center for Clinical Research, Institute for Molecular Cardiovascular Research (IMCAR), RWTH Aachen University, Aachen, Germany
[4] Department of Pathology, Cardiovascular Research Institute Maastricht (CARIM), Maastricht University Medical Centre, Maastricht, The Netherlands
[5] Institute for Cardiovascular Prevention (IPEK), Ludwig-Maximilians-University Munich, Munich, Germany
[6] DZHK (German Centre for Cardiovascular Research), partner site Munich Heart Alliance, Munich, Germany
[7] Inserm UMR 1087, Nantes, France and Nantes Université, CNRS, INSERM, l’institut du thorax, F-44000 Nantes, France
[8] ICBMS UMR CNRS 5246, Université Claude Bernard Lyon 1, France.
[9] Department of Vascular Surgery, Vascular Center, Groupe Hospitalier Paris St Joseph, Paris, France
[10] Department of Nephrology and Clinical Immunology, University Hospital RWTH Aachen
[11] Department of Internal Medicine, Nephrology and Transplantation, Erasmus Medical Center, Rotterdam, The Netherlands
[12] Helmholtz Institute for Biomedical Engineering, Biointerface Lab, RWTH Aachen University, Aachen, Germany
[13] PHY-OS Laboratory, INSERM UMR 1238, Nantes University of Medicine, Nantes, France.

1 Supplementary Figures

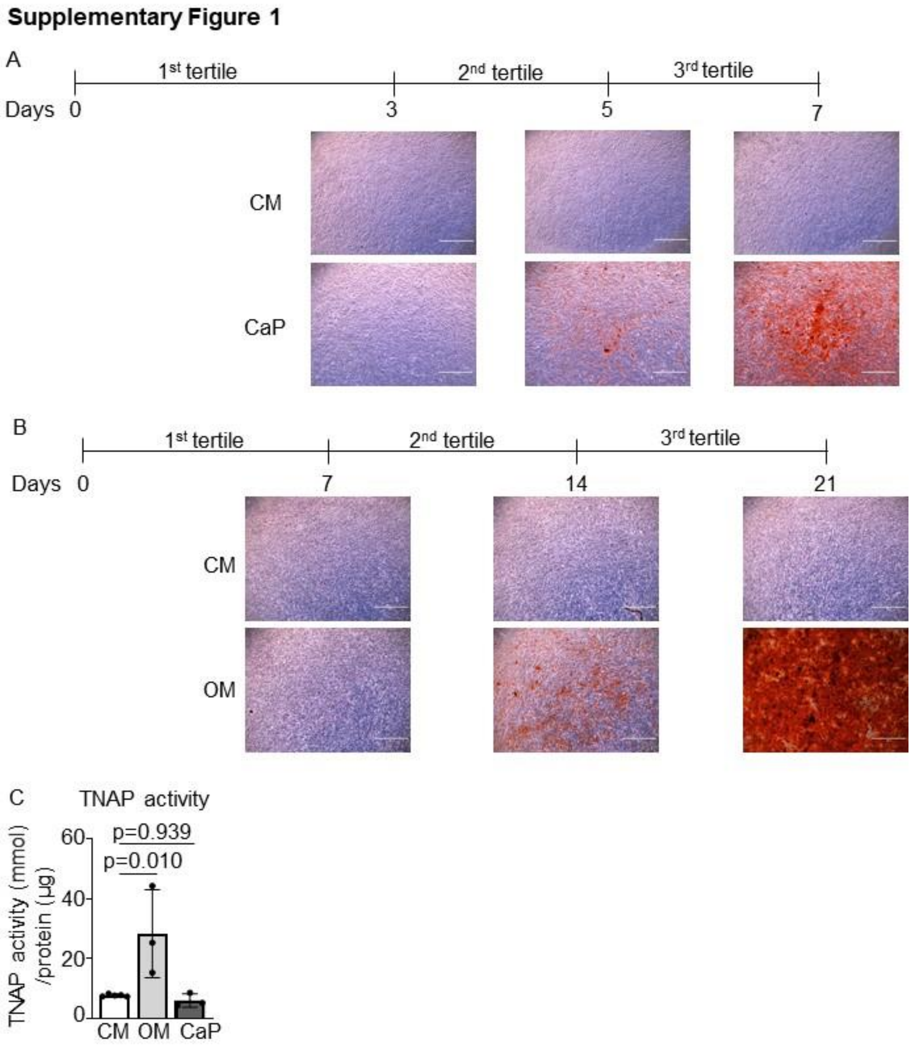

Supplementary Figure 1. Kinetics of matrix mineralization of osteogenic medium (OM) and calcium phosphate (CaP) calcified primary coronary artery smooth muscle cells (pSMCs). (A, B) Representative images of alizarin red staining on days 3, 5, and 7 for CaP (A) and days 7, 14, and 21 for OM (B). Scale bar: 1,000 µm. n = 3. **(C)** Tissue non-specific alkaline phosphatase (TNAP) activity on day 14**.** n = 3 independent pSMC donors. Error bars indicate ± SD. One-way ANOVA with Dunnet's post hoc test.

Supplementary Figure 2

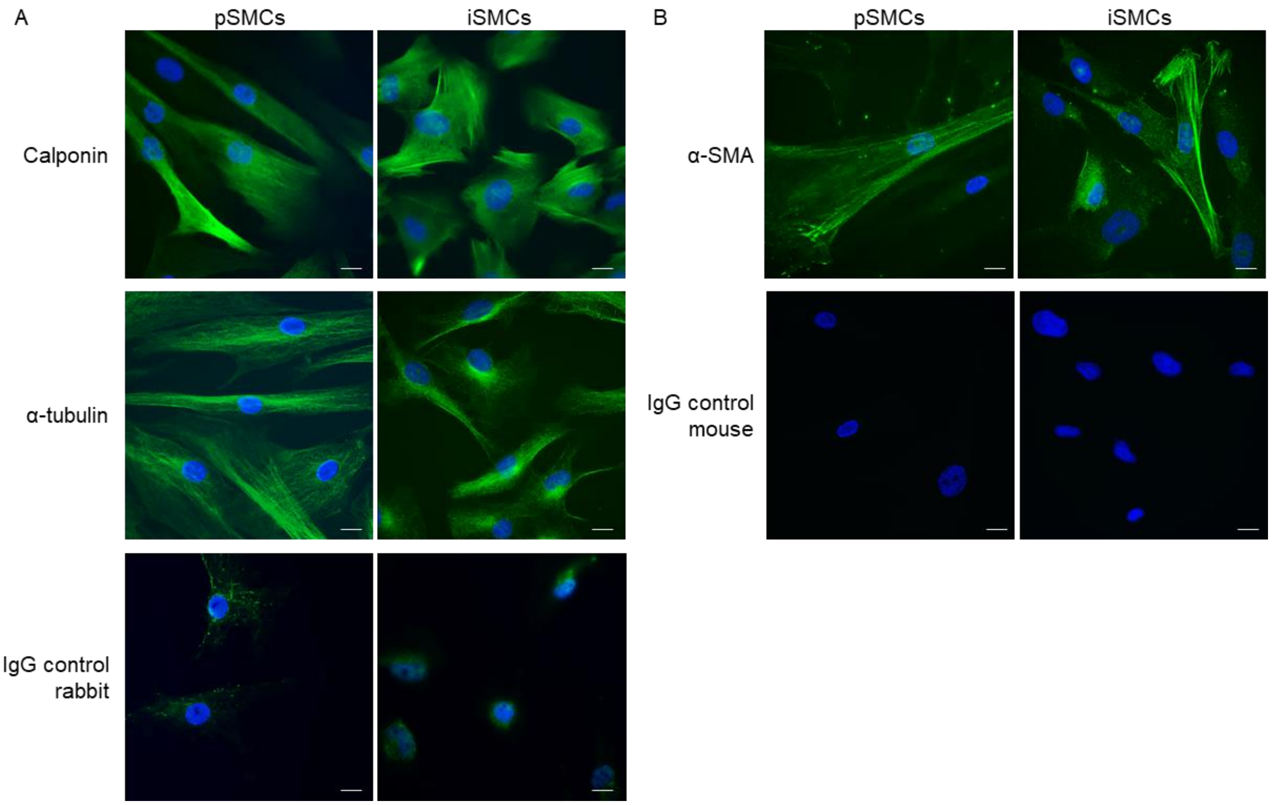

Supplementary Figure 2. Expression of calponin, alpha-tubulin (α-tubulin), and alpha-smooth muscle cell actin (α-SMA) in primary coronary artery smooth muscle cells (pSMCs) and immortalized vascular smooth muscle cells (iSMCs). Representative immunofluorescence images for A) calponin, α-tubulin (rabbit IgG served as a control) and B) α-SMA (mouse-IgG served as a control) (green) and nuclei DAPI staining (blue). (Scale bar: 10 µm. *n* = 3. For iSMCs, n indicates an independent replicate, and for pSMCs an independent cell donor.

Supplementary Figure 3

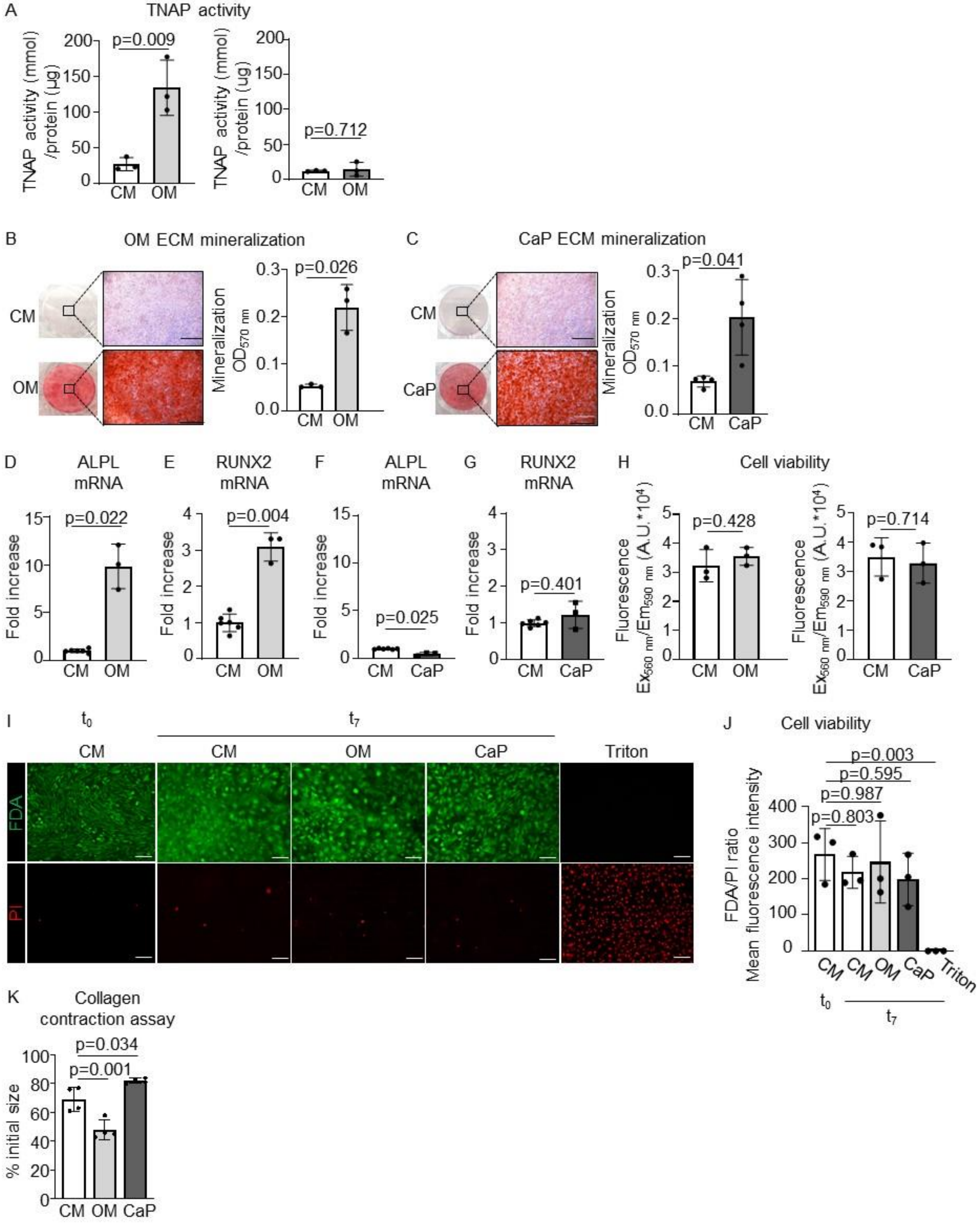

Supplementary Figure 3. Characterization of osteogenic medium (OM) and calcium phosphate (CaP)-induced calcification of immortalized vascular smooth muscle cells (iSMCs). iSMCs were cultured in control medium (CM) and OM for up to 14 days or CaP for up to 7 days. **(A)** TNAP activity

on day 7**.** n = 3. **(B, C)** Representative images of extracellular matrix (ECM) mineralization detected by alizarin red S–stained mineralized matrix and quantification of eluted staining (OM: day 14; CaP: day 7). Scale bar: 1000 µm. n = 3-4. **(D, E)** ALPL and RUNX2 mRNA expression for OM-calcified iSMCs (day 7) and **(F, G)** for CaP-calcified iSMCs (day 3). n = 3. **(H)** Effect of OM and CaP-induced calcification on cell viability measured by Alamar blue assay (Fluorescence Ex_{560} nm/ Em_{590} nm) on day 14 (OM) and day 7 (CaP). n = 3. **(I)** Representative images of live/dead staining (t_0 and day 7 (t_7)). Live cells are stained with fluorescein diacetate (FDA, green) and dead cells with propidium iodide (PI, red). iSMCs were treated with Triton X-100 as a positive control for cell death. Scale bar: 75 µm. **(J)** Quantification of I. FDA/PI mean fluorescence intensity ratio at t_0 and day 7 (t_7). **(K)** Collagen contraction assay at day 5. n = 4. Error bars indicate ± SD. Each n indicates an independent replicate. Unpaired t-test (A-H) and one-way ANOVA with Dunnett's post hoc test (J, K).

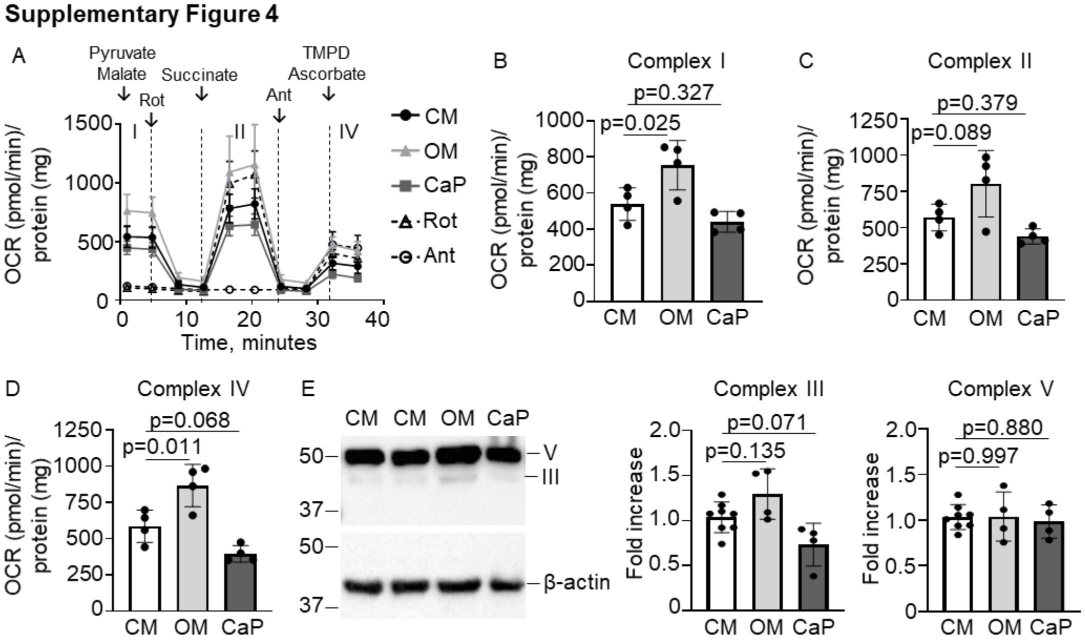

Supplementary Figure 4. Oxygen consumption rate (OCR) measurements for detailed analysis of mitochondria respiratory chain complexes in osteogenic medium (OM) and calcium phosphate (CaP) calcified immortalized vascular smooth muscle cells (iSMCs). (A) Extracellular efflux measurement of OCR over time in response to substrates of respiratory chain complexes added sequentially (pyruvate/ malate; rotenone (Rot); succinate; antimycin A (Ant); tetramethyl-*p*-phenylene diamine (TMPD)/ ascorbate). **(B-D)** Complexes I, II, and IV were analyzed by extracellular efflux analysis. OCR was normalized to protein content. **(E)** Protein expression of the complexes V and III using the OXPHOS antibody cocktail to detect the representative components of complex III - ubiquinol-cytochrome C reductase core protein 2 and complex V - ATP synthase F1 subunit alpha simultaneously. β-actin was used as the loading control. Western blot representative image and quantification. n = 4. Error bars indicate ± SD. Each n indicates an independent replicate. One-way ANOVA with Dunnet’s post hoc test.

Supplementary Figure 5

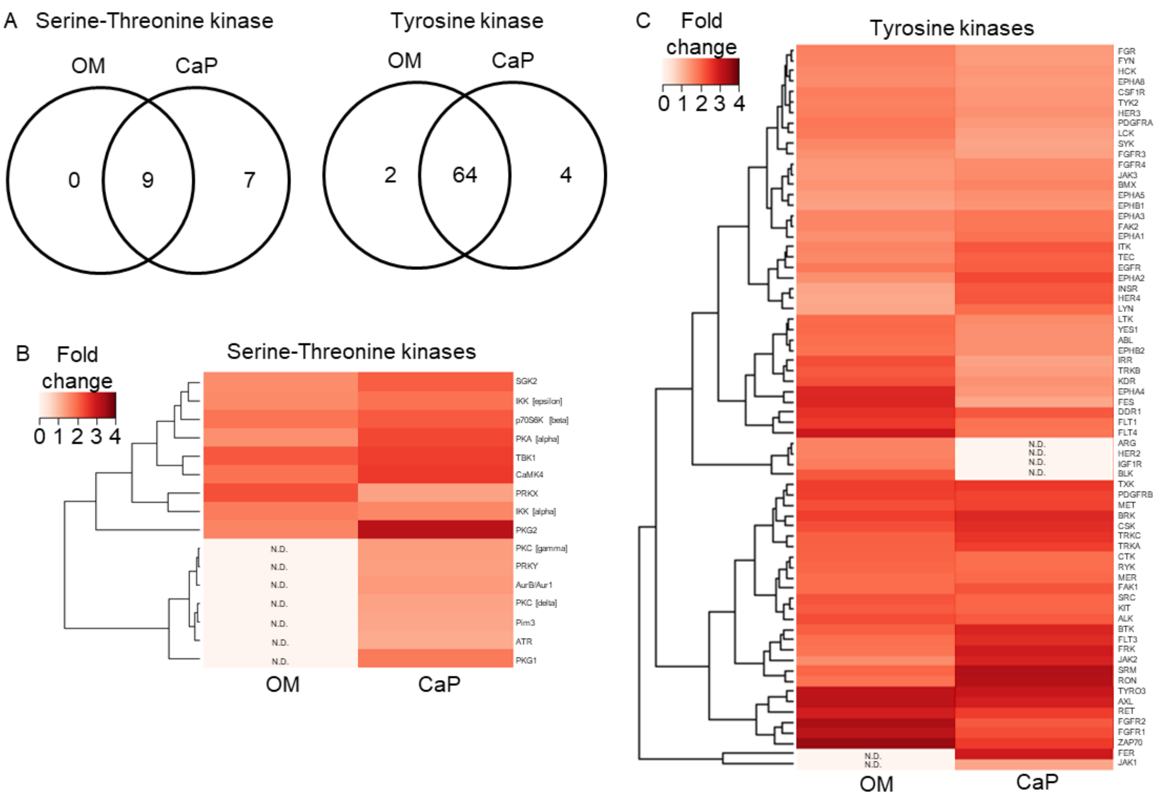

Supplementary Figure 5. Human phospho-kinase array analysis of osteogenic medium (OM) and calcium phosphate (CaP)-calcified immortalized vascular smooth muscle cells (iSMCs). Kinome analysis of serine-threonine kinases (STK) and tyrosine kinases (PTK) was carried out by the peptide array-based kinase activity assay in OM and CaP-treated iSMCs for 24 h. **(A)** Venn diagram of the commonly regulated kinases between OM and CaP-calcified iSMCs. **(B)** Summary heatmap displaying STK and **(C)** PTK with significantly altered activity. n = 3. Each n indicates an independent replicate. N.D.; not determined.

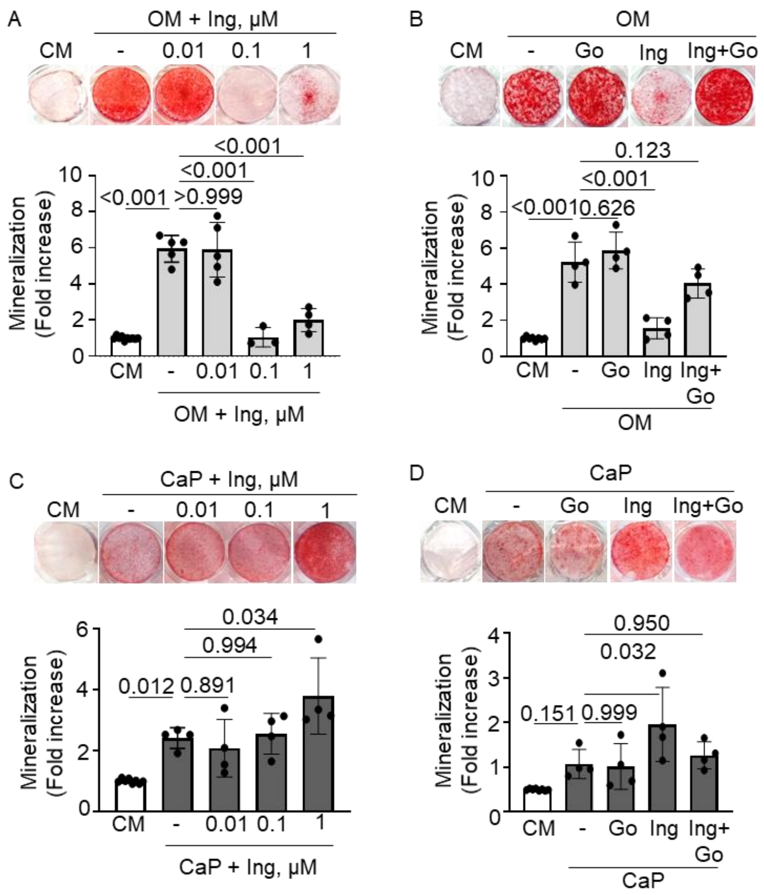

Supplementary Figure 6. Validation of the top drug candidate ingenol in osteogenic medium (OM) and calcium phosphate (CaP) calcified immortalized vascular smooth muscle cell (iSMCs). iSMCs were culture in control medium (CM) and OM for 14 days or CaP for 7 days. Effect of the protein kinase C (PKC) activator ingenol (Ing, 0.01, 0.1, 1.0 µM) and the PKC inhibitor Go6983 (Go) in OM **(A, B)** and CaP **(C, D)**-calcified iSMCs. Go (100 nM) was combined with 1 µM ingenol (B, D). Representative images of extracellular matrix (ECM) mineralization (top) detected by alizarin red S staining and quantification of eluted staining (bottom). n = 3 - 5. DMSO (1:1,000) was used as solvent control in CM, OM, and CaP groups. Error bars indicate ± SD. Each n indicates an independent replicate. One-way ANOVA with Dunnett's post hoc test.

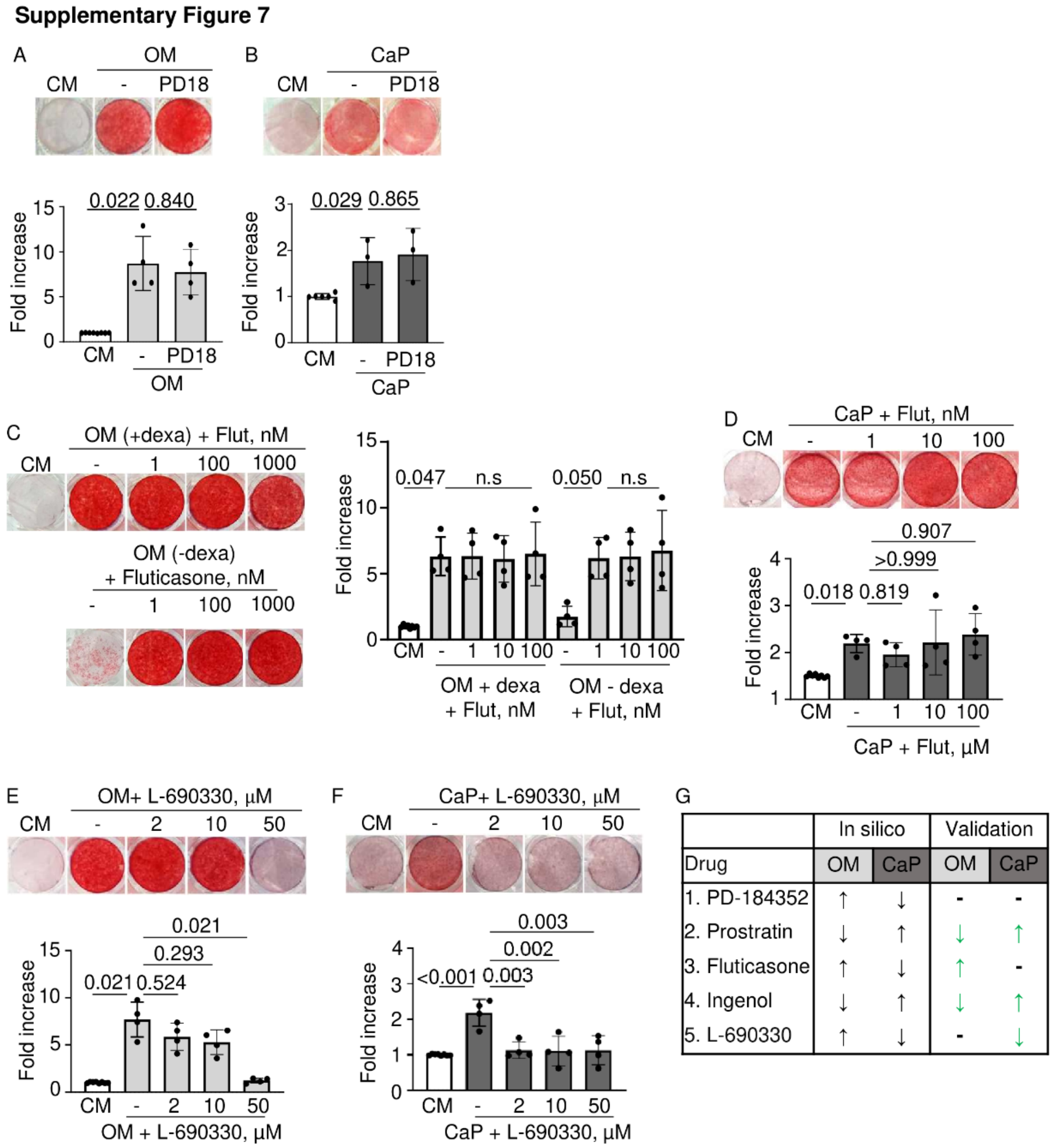

Supplementary Figure 7. Validation of the top drug candidates from osteogenic medium (OM) and calcium phosphate (CaP) calcified immortalized vascular smooth muscle cells (iSMCs). iSMCs were culture in control medium (CM) and OM for 14 days or CaP for 7 days, and calcification was accessed through alizarin red staining visualization and quantification. **(A, B)** The effect of PD184352 (PD18, 1 µM) in (A) OM and (B) CaP-calcified iSMCs. **(C, D)** iSMC cells were treated with fluticasone (Flut; 1, 10, 100 nM) in the presence of OM containing 10 nM dexamethasone (+dexa) or in the absence of it (-dexa) (C) or CaP (D). **(E, F)** The effect of L-690330 (2, 10, 50 µM) in (E) OM and (F) CaP-calcified iSMCs. DMSO (1:1,000; for PD18, Flut) or water (for L-690330) was used as solvent control in CM, OM, and CaP groups. **(G)** Summary of in vitro validation of the top 5 compounds from the in silico drug repurposing analysis. Green arrows indicate a match between the in silico prediction and the in vitro validation. n = 3 - 4. Error bars indicate ±SD. Each n indicates an independent replicate. One-way ANOVA with Dunnett's post hoc test.

Supplementary Figure 8

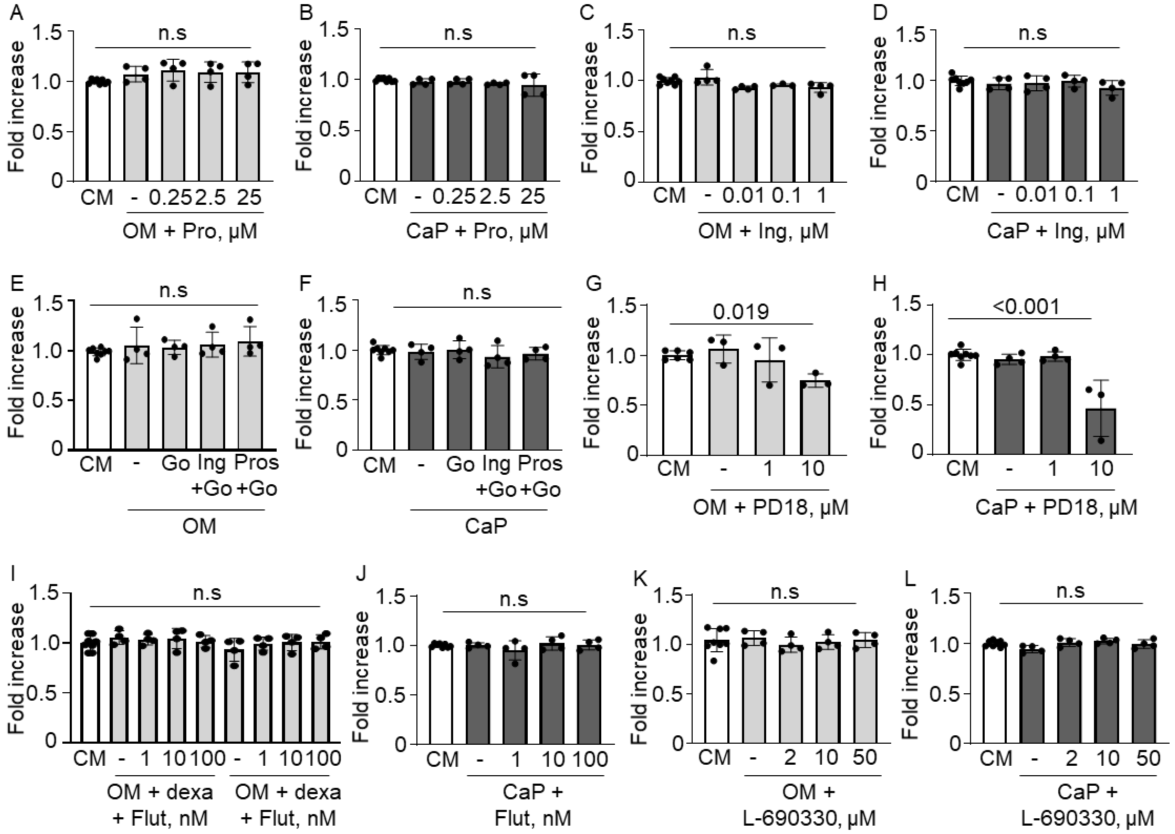

Supplementary Figure 8. Cell viability evaluation of immortalized vascular smooth muscle cells (iSMCs) treated with different compounds from the drug repurposing analysis. AlamarBlue Assay was performed at the endpoint of the calcification assays on day 14 for osteogenic medium (OM) and day 7 for calcium phosphate (CaP)-calcified iSMCs. **(A-D)** Different concentrations of prostratin (Pros) **(A, B)** and ingenol (Ing) **(C, D)** were tested in OM and CaP-calcified iSMCs. **(E, F)** Go6983 (Go, 100 nM) was combined with ingenol (1 µM). **(G, H)** PD184352 (PD18), **(I)** fluticasone (Flut) in the presence of OM containing 100 nM dexamethasone (+dexa) or in the absence of it (-dexa) or **(J)** CaP. **(K, L)** L-690330 was tested using different concentrations. n = 4. DMSO (1:1,000; for Pros, Go, PD18, Flut, Ing) or water (for L-690,330)) was used as solvent control in CM, OM, and CaP groups. Error bars indicate ± SD. Each n indicates an independent replicate. One-way ANOVA with Dunnett's post hoc test.

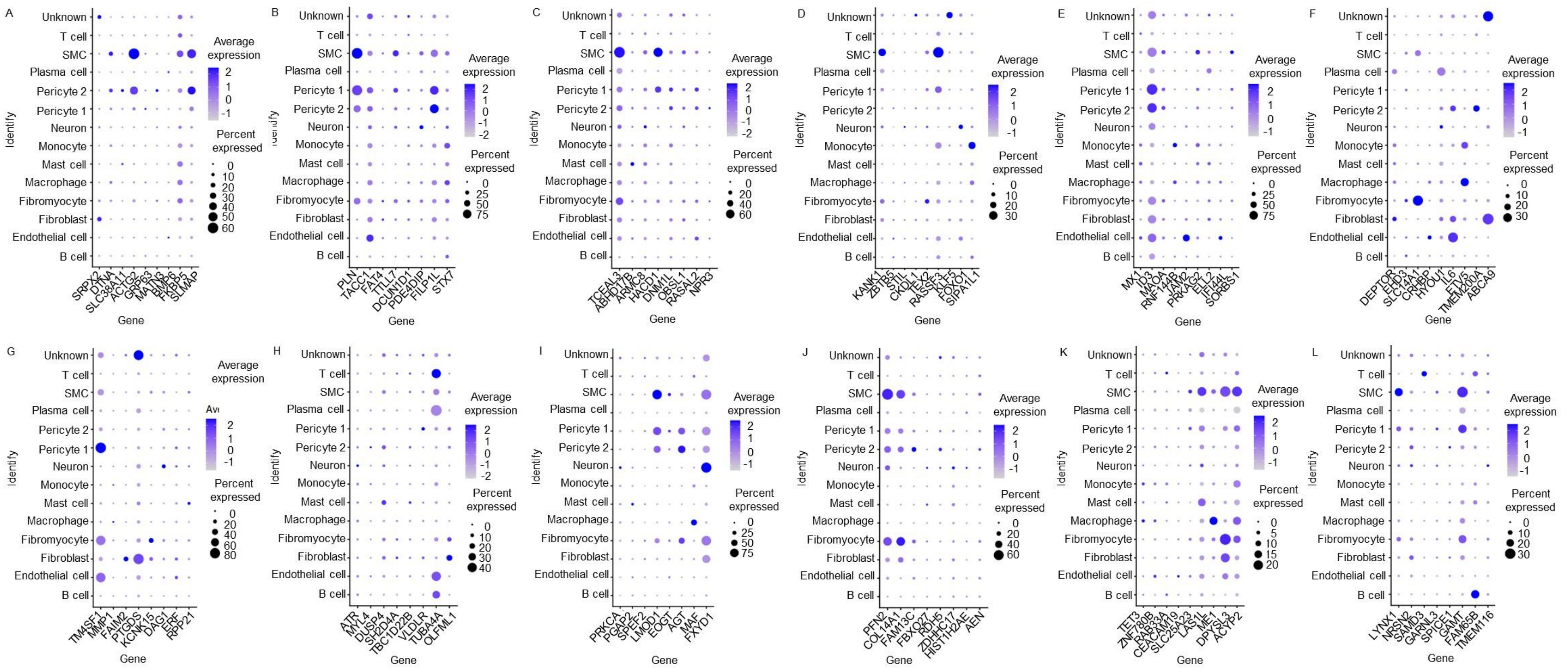

Supplementary Figure 9. Expression dot plots for the genes that were common between osteogenic medium (OM) and calcium phosphate (CaP)-calcified pSMCs queried with the single-cell RNAseq data set from coronary artery (GEO GSE131778; Wirka et al. 2019) using the web-based tool PlaqView. Cell clusters are based on the published annotation (Wirka et al. 2019; Supplemental Fig. 5). 102 out of the 107 genes were identified (not identified: INA, OR2T3, CPA4, VTRNA2, TAS2R19, TAS2R10). The order of genes is based on the ranking from the heat map in Figure 2B. PlaqView allows the query of 9 genes at once. The size of the circles represents the percentage expressed relative in each panel.

Supplementary Figure 10

A

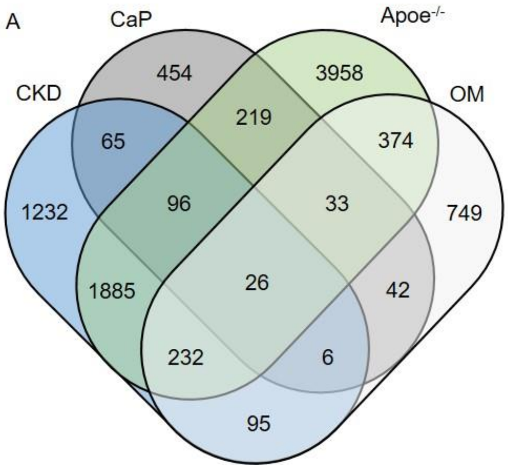

B

% of OM and CaP genes contained in in vivo models*	OM	CaP
Apoe$^{-/-}$	42.7%	39.7%
CKD	23.0%	20.4%

*Differences in Apoe$^{-/-}$ and CKD % might be due to differences in the overall gene number.

Supplementary Figure 10. Comparative transcriptome analysis across mouse and in vitro models. (A) Venn diagram comparing differentially regulated genes (Log_2 FC < -0.5 or > 0.5; $p < 0.05$) in the aorta of Apoe-deficient mice (*Apoe*$^{-/-}$; model of intimal calcification) and chronic kidney disease (CKD) mouse model (5/6 nephrectomy; medial calcification) (GEO GSE159833), with the in vitro SMC calcification models osteogenic medium (OM) and calcium phosphate (CaP). **(B)** Percentage of differentially regulated OM and CaP genes contained in the *Apoe*$^{-/-}$ and CKD mouse model.

2 Supplementary Tables

Supplementary Table 1. TaqMan probes used for real-time PCR.

Gene	Species	Probe
RPLP0	Human	Hs99999902_m1
RUNX2	Human	Hs01047978_m1
ALPL	Human	Hs01029144_m1

Supplementary Table 2. List of differentially regulated genes (FC 1.5) from OM and CaP-calcified pSMCs compared to control (CM). see Excel file

Supplementary Table 3. Over-representation analysis of regulated genes in osteogenic media (OM) at day 7.

Pathway name	Set size	Candidates contained	p-value	q-value	Pathway source
Elastic fibre formation	36	12 (33.3%)	8.5e-5	2.2e-2	Reactome
Regulation of insulin-like growth factor transport and uptake by insulin-like growth factor binding proteins	127	24 (19.0%)	8.2e-5	5.1e-2	Reactome
Molecules associated with elastic fibres	31	10 (32.3%)	1.2ee-4	5.1e-2	Reactome
Metabolism	1972	202 (10.3%)	3e-4	8.6-2	Reactome
Hypertrophic cardiomyopathy	83	17 (20.5%)	3.7e-4	8.6-2	KEGG
Pathogenic Escherichia coli infection	55	13 (23.6%)	4.2e-4	8.6-2	KEGG
Circadian rhythm	31	9 (30.0%)	5e-4	8.6-2	KEGG
Toxicity of botulinum toxin type G	3	3 (100.0%)	5.6e-4	8.6-2	Reactome
Fluid shear stress and atherosclerosis	139	23 (16.5%)	1e-3	0.1	KEGG
Extracellular matrix organization	294	40 (13.6%)	1.1e-3	0.1	Reactome
Sema3A PAK dependent Axon repulsion	16	6 (37.5%)	1.2e-3	0.1	Reactome
Post-translational protein phosphorylation	110	19 (17.4%)	1.4e-3	0.1	Reactome
Amino acid synthesis and interconversion	35	9 (25.7%)	1.7e-3	0.1	Reactome
Arrhythmogenic right ventricular cardiomyopathy	72	14 (19.4%)	2e-3	0.1	KEGG
Transcriptional activation of cell cycle inhibitor p21	4	3 (75.0%)	2.1e-3	0.1	Reactome
Transcriptional activation of p53 responsive genes	4	3 (75.0%)	2.1e-3	0.1	Reactome
Dilated cardiomyopathy	90	16 (18.0%)	2.3e-3	0.1	KEGG
Cytosolic tRNA aminoacylation	24	7 (29.2%)	2.5e-3	0.1	Reactome
Cellular senescence	160	24 (15.0%)	3e-3	0.1	KEGG
Amino acid transport across the plasma membrane	32	8 (25.0%)	3.7e-3	0.2	Reactome

Glutathione synthesis and recycling	15	5 (35.7%)	4e-3	0.2	Reactome
TP53 regulates transcription of several additional cell death genes whose specific roles in p53-dependent apoptosis remain uncertain	14	5 (35.7%)	4e-3	0.2	Reactome
Basigin interactions	27	7 (26.9%)	4.2e-3	0.2	Reactome
Folate biosynthesis	26	7 (26.9%)	4.2e-3	0.2	KEGG
Toxicity of botulinum toxin type F	5	3 (60.0%)	4.9e-3	0.2	Reactome
Toxicity of botulinum toxin type D	5	3 (60.0%)	4.9e-3	0.2	Reactome
FoxO signaling pathway	132	20 (15.3%)	5.3e-3	0.2	KEGG
Arginine and proline metabolism	50	10 (20.4%)	6e-3	0.2	KEGG
Central carbon metabolism in cancer	65	12 (18.5%)	6.3e-3	0.2	KEGG
PI3K events in ERBB4 signaling	10	4 (40.0%)	6.5e-3	0.2	Reactome
Neurotoxicity of clostridium toxins	10	4 (40.0%)	6.5e-3	0.2	Reactome
Circadian Clock	35	8 (22.9%)	6.6e-3	0.2	Reactome
Thyroid hormone signaling pathway	116	18 (15.5%)	6.6e-3	0.2	KEGG
vRNP Assembly	2	2 (100.0%)	6.8e-3	0.2	Reactome
Metabolism of serotonin	2	2 (100.0%)	6.8e-3	0.2	Reactome
AGE-RAGE signaling pathway in diabetic complications	99	16 (16.2%)	6.9e-3	0.2	KEGG
Signaling by VEGF	100	16 (16.0%)	7.7-3	0.2	Reactome
TP53 regulates transcription of cell cycle Genes	51	10 (19.6%)	8e-3	0.2	Reactome
Sphingolipid signaling pathway	118	18 (15.3%)	8e-3	0.2	KEGG
MET activates PI3K/AKT signaling	6	3 (50.0%)	9.3e-3	0.2	Reactome
Synthesis of Lipoxins (LX)	6	3 (50.0%)	9.3e-3	0.2	Reactome
Depolymerisation of the nuclear lamina	11	4 (36.4%)	9.5e-3	0.2	Reactome
The role of GTSE1 in G2/M progression after G2 checkpoint	11	4 (36.4%)	9.5e-3	0.2	Reactome
Pyruvate metabolism	30	7 (23.3%)	9.7e-3	0.2	Reactome
Metabolism of folate and pterines	17	5 (29.4%)	1e-2	0.2	Reactome
Leukocyte transendothelial migration	112	17 (15.2%)	1e-2	0.2	KEGG
Metabolism of carbohydrates	264	33 (12.5%)	1e-2	0.2	Reactome
Arachidonic acid metabolism	62	11 (18.0%)	1e-2	0.2	Reactome
Non-alcoholic fatty liver disease (NAFLD)	149	21 (14.1%)	1e-2	0.2	KEGG
C-type lectin receptor signaling pathway	104	16 (15.4%)	1.1e-2	0.2	KEGG
Glutamate Neurotransmitter Release Cycle	24	6 (25.0%)	1.1e-2	0.2	Reactome
TP53 Regulates Transcription of Genes Involved in G2 Cell Cycle Arrest	18	5 (27.8%)	1.3e-2	0.3	Reactome

Hyaluronan metabolism	12	4 (33.3%)	1.3e-2	0.3	Reactome
Insulin resistance	107	16 (15.0%)	1.4e-2	0.3	KEGG
Transcriptional Regulation by TP53	374	43 (11.6%)	1.4e-2	0.3	Reactome
Semaphorin interactions	64	11 (17.2%)	1.5e-2	0.3	Reactome
Ferroptosis	40	8 (20.0%)	1.5e-2	0.3	KEGG
CS/DS degradation	7	3 (42.9%)	1.5e-2	0.3	Reactome
APOBEC3G mediated resistance to HIV-1 infection	7	3 (42.9%)	1.5e-2	0.3	Reactome
Phase 1 - inactivation of fast Na+ channels	7	3 (42.9%)	1.5e-2	0.3	Reactome
Glutathione metabolism	56	10 (17.9%)	1.5e-2	0.3	KEGG
Keratan sulfate/keratin metabolism	33	7 (21.2%)	1.6e-2	0.3	Reactome
Cooperation of Prefoldin and TriC/CCT in actin and tubulin folding	33	7 (21.2%)	1.6e-2	0.3	Reactome
CD28 co-stimulation	33	7 (21.2%)	1.6e-2	0.3	Reactome
GABA synthesis, release, reuptake and degradation	19	5 (26.3%)	1.6e-2	0.3	Reactome
HSP90 chaperone cycle for steroid hormone receptors	19	5 (26.3%)	1.6e-2	0.3	Reactome
Axon guidance	175	23 (13.1%)	1.7e-2	0.3	KEGG
Signaling by Activin	13	4 (30.8%)	1.8e-2	0.3	Reactome
TNF signaling pathway	110	16 (14.5%)	1.8e-2	0.3	KEGG
VEGFA-VEGFR2 Pathway	92	14 (15.2%)	1.8e-2	0.3	Reactome
Clathrin-mediated endocytosis	138	19 (13.8%)	1.8e-2	0.3	Reactome
Vesicle-mediated transport	620	66 (10.6%)	1.9e-2	0.3	Reactome
African trypanosomiasis	35	7 (20.6%)	1.9e-2	0.3	KEGG
Serotonin clearance from the synaptic cleft	3	2 (66.7%)	1.9e-2	0.3	Reactome
Toxicity of botulinum toxin type B	3	2 (66.7%)	1.9e-2	0.3	Reactome
Integration of viral DNA into host genomic DNA	3	2 (66.7%)	1.9e-2	0.3	Reactome
Autointegration results in viral DNA circles	3	2 (66.7%)	1.9e-2	0.3	Reactome
Metabolism of vitamin K	3	2 (66.7%)	1.9e-2	0.3	Reactome
Integrin cell surface interactions	67	11 (16.4%)	2e-2	0.3	Reactome
TGF-beta signaling pathway	85	13 (15.3%)	2.1e-2	0.3	KEGG
Membrane Trafficking	582	62 (10.7%)	2.2e-2	0.3	Reactome
Neurotransmitter release cycle	51	9 (17.6%)	2.2e-2	0.3	Reactome
Tryptophan catabolism	14	4 (28.6%)	2.3e-2	0.3	Reactome
TP53 regulates transcription of genes involved in G1 cell cycle arrest	14	4 (28.6%)	2.3e-2	0.3	Reactome
Keratan sulfate biosynthesis	28	6 (21.4%)	2.4e-2	0.3	Reactome
Prefoldin mediated transfer of substrate to CCT/TriC	28	6 (21.4%)	2.4e-2	0.3	Reactome

Amino acid and oligopeptide SLC transporters	52	9 (17.3%)	2.5e-2	0.3	Reactome
Proteoglycans in cancer	201	25 (12.4%)	2.5e-2	0.3	KEGG
Detoxification of reactive oxygen species	36	7 (19.4%)	2.5e-2	0.3	Reactome
G alpha (12/13) signalling events	89	13 (14.9%)	2.6e-2	0.3	Reactome
GABAergic synapse	88	13 (14.8%)	2.8e-2	0.3	KEGG
Signaling by nuclear receptors	195	24 (12.4%)	2.8e-2	0.3	Reactome
Thiamine metabolism	16	4 (26.7%)	3e-2	0.3	KEGG
CD28 dependent PI3K/Akt signaling	22	5 (22.7%)	3e-2	0.3	Reactome
Other types of O-glycan biosynthesis	22	5 (22.7%)	3e-2	0.3	KEGG
Pyruvate metabolism and Citric Acid cycle	54	9 (16.7%)	3.1e-2	0.4	Reactome
Synthesis of 5-eicosatetraenoic acids	9	3 (33.3%)	3.2e-2	0.4	Reactome
PI3K/AKT activation	9	3 (33.3%)	3.2e-2	0.4	Reactome
Metabolism of amino acids and derivatives	342	38 (11.2%)	3.3e-2	0.4	Reactome
Pentose phosphate pathway	30	6 (20.0%)	3.3e-2	0.4	KEGG
Neurophilin interactions with VEGF and VEGFR	4	2 (50.0%)	3.6e-2	0.4	Reactome
Antagonism of Activin by Follistatin	4	2 (50.0%)	3.6e-2	0.4	Reactome
Post-chaperonin tubulin folding pathway	23	5 (21.7%)	3.6e-2	0.4	Reactome
Proximal tubule bicarbonate reclamation	23	5 (21.7%)	3.6e-2	0.4	KEGG
Metabolism of vitamins and cofactors	181	22 (12.4%)	3.7e-2	0.4	Reactome
PI3K events in ERBB2 signaling	16	4 (25.0%)	3.7e-2	0.4	Reactome
Synthesis of Prostaglandins and Thromboxanes	16	4 (25.0%)	3.7e-2	0.4	Reactome
CRMPs in Sema3A signaling	16	4 (25.0%)	3.7e-2	0.4	Reactome
Pyruvate metabolism	39	7 (17.9%)	3.8e-2	0.4	KEGG
Trafficking of AMPA receptors	31	6 (19.4%)	3.8e-2	0.4	Reactome
Glutamate binding, activation of AMPA receptors and synaptic plasticity	31	6 (19.4%)	3.8e-2	0.4	Reactome
Mucin type O-glycan biosynthesis	31	6 (19.4%)	3.8e-2	0.4	KEGG
Pathways in cancer	526	55 (10.5%)	4e-2	0.4	KEGG
Regulation of TP53 activity through phosphorylation	93	13 (14.0%)	4.1e-2	0.4	Reactome
ECM proteoglycans	57	9 (15.8%)	4.3e-2	0.4	Reactome
Synthesis of very long-chain fatty acyl-CoAs	24	5 (20.8%)	4.3e-2	0.4	Reactome
Nicotinate metabolism	25	5 (20.8%)	4.3e-2	0.4	Reactome
Pancreatic cancer	75	11 (14.7%)	4.3e-2	0.4	KEGG

Tryptophan metabolism	40	7 (17.5%)	4.3e-2	0.4	KEGG
Glycine, serine and threonine metabolism	40	7 (17.5%)	4.3e-2	0.4	KEGG
Gap junction degradation	10	3 (30.0%)	4.3e-2	0.4	Reactome
HDL remodeling	10	3 (30.0%)	4.3e-2	0.4	Reactome
Hyaluronan uptake and degradation	10	3 (30.0%)	4.3e-2	0.4	Reactome
Aminoacyl-tRNA biosynthesis	66	10 (15.2%)	4.3e-2	0.4	KEGG
Necroptosis	162	20 (12.3%)	4.5e-2	0.4	KEGG
Trafficking of GluR2-containing AMPA receptors	17	4 (23.5%)	4.6e-2	0.4	Reactome
Transmission across chemical synapses	224	26 (11.7%)	4.6e-2	0.4	Reactome
Colorectal cancer	86	12 (14.0%)	4.9e-2	0.4	KEGG

Supplementary Table 4. Over-representation analysis of regulated genes in calcium phosphate media (CaP) at day 3.

Pathway name	Set size	Candidates contained	p-value	q-value	Pathway source
Phosphatidylinositol signaling system	99	13 (13.1%)	4.7e-4	0.1	KEGG
Regulation of pyruvate dehydrogenase complex	16	5 (31.2%)	5.2e-4	0.1	Reactome
Smooth muscle contraction	35	7 (20.0%)	8e-4	0.1	Reactome
Cellular responses to external stimuli	414	33 (8.0%)	9.8e-4	0.1	Reactome
Prostate cancer	97	12 (12.4%)	1.3e-3	0.1	KEGG
Inositol phosphate metabolism	74	10 (13.5%)	1.6e-3	0.1	KEGG
Pyruvate metabolism	30	6 (20.0%)	1.8e-3	0.1	Reactome
Mitochondrial translation termination	89	11 (12.5%)	1.9e-3	0.1	Reactome
Cilium Assembly	187	18 (9.6%)	1.9e-3	0.1	Reactome
Thyroid hormone signaling pathway	116	13 (11.2%)	2.1e-3	0.1	KEGG
Pyruvate metabolism and Citric Acid cycle	54	8 (14.8%)	2.6e-3	0.2	Reactome
Mitochondrial translation	95	11 (11.7%)	3.3e-3	0.2	Reactome
Anchoring of the basal body to the plasma membrane	97	11 (11.3%)	4.1e-3	0.2	Reactome
BH3-only proteins associate with and inactivate anti-apoptotic BCL-2 members	8	3 (37.5%)	4.2e-3	0.2	Reactome
TGF-beta signaling pathway	85	10 (11.8%)	4.6e-3	0.2	KEGG
HATs acetylate histones	142	14 (9.9%)	4.7e-3	0.2	Reactome
Organelle biogenesis and maintenance	240	20 (8.3%)	5.8e-3	0.2	Reactome
Mitochondrial translation elongation	89	10 (11.4%)	6e-3	0.2	Reactome
Mitochondrial translation initiation	89	10 (11.4%)	6e-3	0.2	Reactome
Insulin signaling pathway	137	13 (9.5%)	8.7e-3	0.3	KEGG
Apelin signaling pathway	137	13 (9.5%)	8.7e-3	0.3	KEGG
Cushing syndrome	154	14 (9.1%)	9.5e-3	0.3	KEGG
Pre-NOTCH transcription and translation	30	5 (16.7%)	1e-2	0.3	Reactome
Pre-NOTCH expression and processing	42	6 (14.3%)	1e-2	0.3	Reactome
NOTCH3 intracellular domain Regulates transcription	20	4 (20.0%)	1.1e-2	0.3	Reactome
Phosphorylation of the APC/C	20	4 (20.0%)	1.1e-2	0.3	Reactome
Interleukin-6 signaling	11	3 (27.3%)	1.1e-2	0.3	Reactome
TET1,2,3 and TDG demethylate DNA	4	2 (50.0%)	1.1e-2	0.3	Reactome
Cellular responses to stress	345	25 (7.3%)	1.2e-2	0.3	Reactome
PI Metabolism	84	9 (10.7%)	1.2e-2	0.3	Reactome

AGE-RAGE signaling pathway in diabetic complications	99	10 (10.1%)	1.3e-2	0.3	KEGG
ECM proteoglycans	57	7 (12.3%)	1.3e-2	0.3	Reactome
Macroautophagy	57	7 (12.3%)	1.3e-2	0.3	Reactome
Metallothioneins bind metals	12	3 (25.0%)	1.4e-2	0.3	Reactome
Muscle contraction	195	16 (8.2%)	1.4e-2	0.3	Reactome
mTOR signaling pathway	151	13 (8.7%)	1.7e-2	0.3	KEGG
Epigenetic regulation of gene expression	151	13 (8.7%)	1.7e-2	0.3	Reactome
Signaling by NOTCH	120	11 (9.2%)	1.8e-2	0.3	Reactome
Renin-angiotensin system	23	4 (17.4%)	1.8e-2	0.3	KEGG
Estrogen biosynthesis	5	2 (40.0%)	1.8e-2	0.3	Reactome
Chromatin modifying enzymes	272	20 (7.4%)	2.1e-2	0.4	Reactome
Chromatin organization	272	20 (7.4%)	2.1e-2	0.4	Reactome
Cellular Senescence	189	15 (8.0%)	2.2e-2	0.4	Reactome
Cell cycle	124	11 (8.9%)	2.3e-2	0.4	KEGG
Recruitment of NuMA to mitotic centrosomes	79	8 (10.1%)	2.5e-2	0.4	Reactome
Renin secretion	65	7 (10.8%)	2.6e-2	0.4	KEGG
Recruitment of mitotic centrosome proteins and complexes	80	8 (10.0%)	2.6e-2	0.4	Reactome
Centrosome maturation	80	8 (10.0%)	2.6e-2	0.4	Reactome
Response to metal ions	15	3 (20.0%)	2.7e-2	0.4	Reactome
Inflammasomes	15	3 (20.0%)	2.7e-2	0.4	Reactome
Mitotic Prophase	144	12 (8.4%)	2.7e-2	0.4	Reactome
Senescence-Associated Secretory Phenotype	112	10 (9.0%)	2.7e-2	0.4	Reactome
Signaling by Nuclear Receptors	195	15 (7.8%)	2.7e-2	0.4	Reactome
Signaling by TGF-beta Receptor Complex	67	7 (10.4%)	3e-2	0.4	Reactome
APC/C:Cdh1 mediated degradation of Cdc20 and other APC/C:Cdh1 targeted proteins in late mitosis/early G1	27	4 (14.8%)	3.1e-2	0.4	Reactome
Signaling by Rho GTPases	435	28 (6.5%)	3.1e-2	0.4	Reactome
Human papillomavirus infection	339	23 (6.8%)	3.2e-2	0.4	KEGG
M Phase	340	23 (6.8%)	3.2e-2	0.4	Reactome
Hypertrophic cardiomyopathy	83	8 (9.6%)	3.2e-2	0.4	KEGG
Regulation of KIT signaling	16	3 (18.8%)	3.2e-2	0.4	Reactome
eNOS activation and regulation	16	3 (18.8%)	3.2e-2	0.4	Reactome
Metabolism of nitric oxide	16	3 (18.8%)	3.2e-2	0.4	Reactome
Synthesis of PIPs at the early endosome membrane	16	3 (18.8%)	3.2e-2	0.4	Reactome
Loss of Nlp from mitotic centrosomes	69	7 (10.1%)	3.4e-2	0.4	Reactome

Loss of proteins required for interphase microtubule organization from the centrosome	69	7 (10.1%)	3.4e-2	0.4	Reactome
Recycling pathway of L1	29	4 (14.3%)	3.5e-2	0.4	Reactome
Transport of nucleosides and free purine and pyrimidine bases across the plasma membrane	7	2 (28.6%)	3.6e-2	0.4	Reactome
Anchoring fibril formation	7	2 (28.6%)	3.6e-2	0.4	Reactome
Non-integrin membrane-ECM interactions	42	5 (11.9%)	3.9e-2	0.4	Reactome
RUNX1 regulates transcription of genes involved in differentiation of HSCs	88	8 (9.3%)	3.9e-2	0.4	Reactome
Energy dependent regulation of mTOR by LKB1-AMPK	29	4 (13.8%)	3.9e-2	0.4	Reactome
Transcriptional misregulation in cancer	186	14 (7.6%)	3.9e-2	0.4	KEGG
Calcium signaling pathway	186	14 (7.6%)	3.9e-2	0.4	KEGG
Regulation of PLK1 Activity at G2/M Transition	87	8 (9.2%)	4.1e-2	0.4	Reactome
tRNA processing in the nucleus	57	6 (10.5%)	4.2e-2	0.4	Reactome
AURKA Activation by TPX2	72	7 (9.7%)	4.2e-2	0.4	Reactome
Activation of BH3-only proteins	30	4 (13.3%)	4.3e-2	0.4	Reactome
Synthesis of PIPs at the Golgi membrane	18	3 (16.7%)	4.4e-2	0.4	Reactome
Ubiquitin mediated proteolysis	137	11 (8.0%)	4.4e-2	0.4	KEGG
Rap1 signaling pathway	206	15 (7.3%)	4.5e-2	0.4	KEGG
Vascular smooth muscle contraction	121	10 (8.3%)	4.5e-2	0.4	KEGG
Bacterial invasion of epithelial cells	74	7 (9.5%)	4.8e-2	0.4	KEGG
The citric acid cycle and respiratory electron transport	173	13 (7.5%)	4.8e-2	0.4	Reactome
Synthesis of active ubiquitin: roles of E1 and E2 enzymes	31	4 (12.9%)	4.8e-2	0.4	Reactome
Mitotic G2-G2/M phases	139	11 (7.9%)	4.8e-2	0.4	Reactome

Supplementary Table 5. Over-representation analysis of 107 common regulated genes in osteogenic media (OM) and calcium phosphate media (CaP).

**Pathway name**	**Set size**	**Candidates contained**	**p-value**	**q-value**	**Pathway source**
Muscle contraction	195	7 (3.6%)	1.4e-4	1.9e-2	Reactome
Smooth Muscle Contraction	35	3 (8.6%)	1.1e-3	6.6e-2	Reactome
EGFR Transactivation by Gastrin	10	2 (20.0%)	1.4e-3	6.6e-2	Reactome
Gastrin-CREB signalling pathway via PKC and MAPK	19	2 (10.5%)	5.4e-3	0.1	Reactome
Signaling by ROBO receptors	66	3 (4.5%)	6.7e-3	0.1	Reactome
Extracellular matrix organization	294	6 (2.0%)	7.4e-3	0.1	Reactome
PPAR signaling pathway	74	3 (4.1%)	9.2e-3	0.1	KEGG
Hypertrophic cardiomyopathy	83	3 (3.6%)	1.2e-2	0.1	KEGG
Fatty acid elongation	30	2 (6.7%)	1.3e-2	0.1	KEGG
African trypanosomiasis	35	2 (5.9%)	1.6e-2	0.1	KEGG
Collagen degradation	35	2 (5.7%)	1.7e-2	0.1	Reactome
AGE-RAGE signaling pathway in diabetic complications	99	3 (3.0%)	2e-2	0.1	KEGG
Pyruvate metabolism	39	2 (5.1%)	2.1e-2	0.1	KEGG
Glycine, serine and threonine metabolism	40	2 (5.0%)	2.2e-2	0.1	KEGG
Class C/3 (Metabotropic glutamate/pheromone receptors)	40	2 (5.0%)	2.2e-2	0.1	Reactome
Transcriptional misregulation in cancer	186	4 (2.2%)	2.3e-2	0.1	KEGG
Insulin resistance	107	3 (2.8%)	2.4e-2	0.1	KEGG
Non-integrin membrane-ECM interactions	42	2 (4.8%)	0.025	0.1	Reactome
Thyroid hormone signaling pathway	116	3 (2.6%)	3e-2	0.2	KEGG
Arginine and proline metabolism)	50	2 (4.1%)	3.3e-2	0.2	KEGG
Cellular responses to external stimuli	414	6 (1.5%)	3.3e-2	0.2	Reactome
Ion homeostasis	52	2 (3.8%)	3.7e-2	0.2	Reactome
Regulation of insulin-like growth factor transport and uptake by insulin-like growth factor binding proteins	127	3 (2.4%)	3.7e-2	0.2	Reactome
Ion transport by P-type ATPases	55	2 (3.6%)	4.1e-2	0.2	Reactome
Pathogenic Escherichia coli infection	55	2 (3.6%)	4.1e-2	0.2	KEGG
FoxO signaling pathway	132	3 (2.3%)	4.1e-2	0.2	KEGG
Cardiac conduction	131	3 (2.3%)	4.1e-2	0.2	Reactome
ECM proteoglycans	57	2 (3.5%)	4.3e-2	0.2	Reactome
Insulin signaling pathway	137	3 (2.2%)	4.6e-2	0.2	KEGG

Supplementary Table 6. Genes that are decreased in the calcified carotid artery and shared with dysregulated genes in osteogenic medium (OM), calcium phosphate (CaP), or both.

Dysregulated in		
OM	CaP	OM and CaP
ABI3BP	AGTR1	ACTG2
ADAM32	AKAP12	ELL2
ADD3	AR	FXYD1
ADH1B	ARHGEF25	ID3
ADRA1D	BAMBI	JAM2
AKAP2	BEND5	KANK1
ALDH1B1	CASQ2	LMOD1
ALDH1L1	CDKL5	MAOA
ANGPTL1	CNN1	NPR3
ANK2	COX7A1	PLN
AOX1	CSRP2	RASSF3
APBB2	DIXDC1	RDH5
APCDD1	DLG5	SLC25A23
ARHGEF26	FGFR1	SLMAP
ARHGEF9	FOXN3	SORBS1
ARID5A	HEYL	TTLL7
ASNS	HIST1H1C	
ASPA	ID2	
ATP8B1	ID4	
CACNB1	ITGA7	
CAMK2N1	KCNAB1	
CDH19	KCNJ8	
CERS1	KLHL13	
CITED4	LGI4	
CLMP	LRIG3	
CLSTN2	LRRC8A	
CLU	MANSC1	
CRISPLD2	MAP2	
CTNNAL1	MMP16	
DGKD	MPP6	
DIAPH2	MUSTN1	
DNAJB4	MYH11	
ELN	MYOM1	
ERRFI1	NDN	
FAM110B	NLGN1	
FAM129A	NPR1	
FBLIM1	OTUD7B	
FERMT2	PALM	
FHL5	PDLIM3	
FIBIN	PHLDB1	
FRY	PLCB4	
FSTL3	PLCE1	
GJA1	PPP1R3B	

GNG11	PRICKLE1
GPRC5A	PRUNE2
H2AFJ	RAB23
HEY2	REEP2
HSPA2	SNTA1
HSPB8	SSPN
IL17RD	TCF7L1
IL31RA	TGFB3
IMMP2L	TMEM61
IRS2	TRPC4
ITGA8	TXNDC15
KCND3	
KCNIP3	
KCNMA1	
KIF1C	
KLF9	
LAMA2	
LARP6	
LAYN	
LMF1	
LPAR1	
LRP6	
MAP6	
MBNL2	
MFAP3L	
MYOZ2	
NAP1L5	
NEXN	
NFIA	
NFYB	
OSR1	
PCBP4	
PCDHB11	
PDE1C	
PDE4D	
PELI2	
PHGDH	
PKD2	
POLR1E	
PPL	
PPP1CB	
PPP1R14A	
PRKCDBP	
PRKD1	
PRRX1	
PSIP1	
PTGR1	
PYGM	

RAVER2
RBPMS
ROR1
RRAS2
SCRG1
SELM
SEMA3D
SGCD
SH3BGR
SH3D19
SLC24A3
SLIT3
SMAD3
SOCS2
SPARCL1
SPG20
SRPX
STAC
STK32A
SYNE2
TBC1D1
TC2N
TCEA3
TCEAL1
TCEAL4
TCEAL6
TFPI2
TGFBR3
TRIM2
USP53
ZBTB16
ZBTB47
ZHX3
ZNF483

Supplementary Table 7. Genes that are increased in the calcified carotid artery and shared with dysregulated genes in osteogenic medium (OM), calcium phosphate (CaP), or both.

Dysregulated in		
OM	CaP	OM and CaP
ABCA7	ACOT13	CRHBP
ADAM12	ANPEP	DEPTOR
ALDOA	APBB1IP	ETV5
ALOX5AP	ARRB1	IFI44L
AP2S1	BBC3	MAF
APOE	BHLHE41	MMP1
ARL4C	CCDC30	MX1
ARSB	CENPBD1	PRKCA
ATG16L2	CHCHD10	TMEM200A
ATP6AP1	CYB561D2	
ATP6V0D1	DFNA5	
BCAT1	ECEL1P2	
BLM	ENC1	
CARD16	EPSTI1	
CDK1	FASTKD1	
CERS6	FKBP15	
CHST15	FLVCR2	
CLN6	GAL3ST4	
CMTM7	GRAMD1B	
COL5A1	HK3	
COMMD9	HPCAL1	
COTL1	IFI6	
CTSD	IFIH1	
CTSK	IGSF6	
CXCL16	LST1	
DAB2	MYO5A	
DBP	NETO2	
DENND4B	OAS2	
DPP4	OPN3	
EGR2	PARP15	
ERMP1	PFKFB2	
FABP5	PI4K2A	
FAP	PPFIBP2	
FCGR2A	PYCARD	
FMNL2	RCAN3	
FNDC1	RRAGD	
FST	RSPH3	
GALNT1	SGK223	
GINS2	SH3BP2	
GNPTAB	SLC25A19	
GPNMB	SLC29A3	
GPX1	SLC6A8	
HEXB	TDP2	
HLA-DPB1	TMEM206	

HLA-G	TNFAIP8
HMGA1	TPI1P2
HPGD	TRAF3IP3
IL18	UHRF1BP1
ITGA4	UNC93B1
JAK3	
LACC1	
LGALS3	
LUM	
LY96	
LYPD1	
M6PR	
MAPK13	
MIAT	
MOXD1	
MPDU1	
MYBPH	
NDUFA4L2	
NRP2	
NTM	
NXPH4	
P4HA1	
PAK1	
PGD	
PLAU	
PLXNC1	
PODXL	
PTK2B	
RAB11FIP1	
RAP2B	
RASSF4	
RIN3	
SAMHD1	
SECTM1	
SLAMF6	
SLC39A4	
SLC9A3R1	
SNX11	
SRGAP3	
ST3GAL6	
STAT1	
TGFBI	
TM6SF1	
TMEM91	
TNFSF13B	
TPI1	
TPX2	

TUBB3
UNC5B
VSNL1
ZNF385A

Supplementary Table 8. The presence of genes common between osteogenic medium (OM) and calcium phosphate (CaP)-calcified primary coronary artery smooth muscle cells (pSMCs) in cell clusters from human coronary artery (GEO GSE131778; Wirka et al. 2019) using the web-based tool PlaqView. Cell clusters are based on the published annotation (Wirka et al. 2019; Supplemental Fig. 5). Only genes that were present in at least 20 % of the cells are shown. Cell clusters with the highest expression are marked in green.

<table>
<tr><th></th><th>SMC</th><th>Pericyte 1</th><th>Fibro-myocyte</th><th>Fibro-blast</th><th>Endo-thelial</th><th>Plasma cell</th><th>Neuron</th><th>Mono-cyte</th><th>Un-known</th><th>Pericyte 2</th><th>Macro-phage</th><th>Mast cell</th><th>B cell</th></tr>
<tr><td>SLMAP</td><td style="background:#c6e0b4"></td><td></td><td></td><td></td><td></td><td></td><td></td><td></td><td></td><td></td><td></td><td></td><td></td></tr>
<tr><td>MAOA</td><td style="background:#c6e0b4"></td><td></td><td></td><td></td><td></td><td></td><td></td><td></td><td></td><td></td><td></td><td></td><td></td></tr>
<tr><td>FKBP5</td><td style="background:#c6e0b4"></td><td></td><td></td><td></td><td></td><td></td><td></td><td></td><td></td><td></td><td></td><td></td><td></td></tr>
<tr><td>HACD1</td><td style="background:#c6e0b4"></td><td></td><td></td><td></td><td></td><td></td><td></td><td></td><td></td><td></td><td></td><td></td><td></td></tr>
<tr><td>TCEAL3</td><td style="background:#c6e0b4"></td><td></td><td></td><td></td><td></td><td></td><td></td><td></td><td></td><td></td><td></td><td></td><td></td></tr>
<tr><td>KANK1</td><td style="background:#c6e0b4"></td><td></td><td></td><td></td><td></td><td></td><td></td><td></td><td></td><td></td><td></td><td></td><td></td></tr>
<tr><td>RASSF3</td><td style="background:#c6e0b4"></td><td></td><td></td><td></td><td></td><td></td><td></td><td></td><td></td><td></td><td></td><td></td><td></td></tr>
<tr><td>ACYP2</td><td style="background:#c6e0b4"></td><td></td><td></td><td></td><td></td><td></td><td></td><td></td><td></td><td></td><td></td><td></td><td></td></tr>
<tr><td>PLN</td><td style="background:#c6e0b4"></td><td></td><td></td><td></td><td></td><td></td><td></td><td></td><td></td><td></td><td></td><td></td><td></td></tr>
<tr><td>ARMC8</td><td style="background:#c6e0b4"></td><td></td><td></td><td></td><td></td><td></td><td></td><td></td><td></td><td></td><td></td><td></td><td></td></tr>
<tr><td>PFN2</td><td style="background:#c6e0b4"></td><td></td><td></td><td></td><td></td><td></td><td></td><td></td><td></td><td></td><td></td><td></td><td></td></tr>
<tr><td>TTLL7</td><td style="background:#c6e0b4"></td><td></td><td></td><td></td><td></td><td></td><td></td><td></td><td></td><td></td><td></td><td></td><td></td></tr>
<tr><td>LMOD1</td><td style="background:#c6e0b4"></td><td></td><td></td><td></td><td></td><td></td><td></td><td></td><td></td><td></td><td></td><td></td><td></td></tr>
<tr><td>GAMT</td><td style="background:#c6e0b4"></td><td></td><td></td><td></td><td></td><td></td><td></td><td></td><td></td><td></td><td></td><td></td><td></td></tr>
<tr><td>PRKAG2</td><td style="background:#c6e0b4"></td><td></td><td></td><td></td><td></td><td></td><td></td><td></td><td></td><td></td><td></td><td></td><td></td></tr>
<tr><td>SORBS1</td><td style="background:#c6e0b4"></td><td></td><td></td><td></td><td></td><td></td><td></td><td></td><td></td><td></td><td></td><td></td><td></td></tr>
<tr><td>LAS1L</td><td style="background:#c6e0b4"></td><td></td><td></td><td></td><td></td><td></td><td></td><td></td><td></td><td></td><td></td><td></td><td></td></tr>
<tr><td>LYNX1</td><td style="background:#c6e0b4"></td><td></td><td></td><td></td><td></td><td></td><td></td><td></td><td></td><td></td><td></td><td></td><td></td></tr>
<tr><td>ACTG2</td><td style="background:#c6e0b4"></td><td></td><td></td><td></td><td></td><td></td><td></td><td></td><td></td><td></td><td></td><td></td><td></td></tr>
<tr><td>EOGT</td><td style="background:#c6e0b4"></td><td></td><td></td><td></td><td></td><td></td><td></td><td></td><td></td><td></td><td></td><td></td><td></td></tr>
<tr><td>TMEM200A</td><td></td><td style="background:#c6e0b4"></td><td></td><td></td><td></td><td></td><td></td><td></td><td></td><td></td><td></td><td></td><td></td></tr>
<tr><td>AGT</td><td></td><td style="background:#c6e0b4"></td><td></td><td></td><td></td><td></td><td></td><td></td><td></td><td></td><td></td><td></td><td></td></tr>
<tr><td>FILIP1L</td><td></td><td style="background:#c6e0b4"></td><td></td><td></td><td></td><td></td><td></td><td></td><td></td><td></td><td></td><td></td><td></td></tr>
<tr><td>TM4SF1</td><td></td><td style="background:#c6e0b4"></td><td></td><td></td><td></td><td></td><td></td><td></td><td></td><td></td><td></td><td></td><td></td></tr>
<tr><td>FAM13C</td><td></td><td style="background:#c6e0b4"></td><td></td><td></td><td></td><td></td><td></td><td></td><td></td><td></td><td></td><td></td><td></td></tr>
<tr><td>COL14A1</td><td></td><td></td><td style="background:#c6e0b4"></td><td></td><td></td><td></td><td></td><td></td><td></td><td></td><td></td><td></td><td></td></tr>
<tr><td>SLC14A1</td><td></td><td></td><td style="background:#c6e0b4"></td><td></td><td></td><td></td><td></td><td></td><td></td><td></td><td></td><td></td><td></td></tr>
<tr><td>DPYSL3</td><td></td><td></td><td style="background:#c6e0b4"></td><td></td><td></td><td></td><td></td><td></td><td></td><td></td><td></td><td></td><td></td></tr>
<tr><td>SRPX2</td><td></td><td></td><td></td><td style="background:#c6e0b4"></td><td></td><td></td><td></td><td></td><td></td><td></td><td></td><td></td><td></td></tr>
<tr><td>FAIM2</td><td></td><td></td><td></td><td style="background:#c6e0b4"></td><td></td><td></td><td></td><td></td><td></td><td></td><td></td><td></td><td></td></tr>
<tr><td>OLFML1</td><td></td><td></td><td></td><td style="background:#c6e0b4"></td><td></td><td></td><td></td><td></td><td></td><td></td><td></td><td></td><td></td></tr>
<tr><td>IL6</td><td></td><td></td><td></td><td></td><td style="background:#c6e0b4"></td><td></td><td></td><td></td><td></td><td></td><td></td><td></td><td></td></tr>
<tr><td>TACC1</td><td></td><td></td><td></td><td></td><td style="background:#c6e0b4"></td><td></td><td></td><td></td><td></td><td></td><td></td><td></td><td></td></tr>
<tr><td>IFI44L</td><td></td><td></td><td></td><td></td><td style="background:#c6e0b4"></td><td></td><td></td><td></td><td></td><td></td><td></td><td></td><td></td></tr>
<tr><td>JAM2</td><td></td><td></td><td></td><td></td><td style="background:#c6e0b4"></td><td></td><td></td><td></td><td></td><td></td><td></td><td></td><td></td></tr>
<tr><td>HYOU1</td><td></td><td></td><td></td><td></td><td></td><td style="background:#c6e0b4"></td><td></td><td></td><td></td><td></td><td></td><td></td><td></td></tr>
<tr><td>TUBA4A</td><td></td><td></td><td></td><td></td><td></td><td style="background:#c6e0b4"></td><td></td><td></td><td></td><td></td><td></td><td></td><td></td></tr>
<tr><td>ELL2</td><td></td><td></td><td></td><td></td><td></td><td style="background:#c6e0b4"></td><td></td><td></td><td></td><td></td><td></td><td></td><td></td></tr>
<tr><td>DAG1</td><td></td><td></td><td></td><td></td><td></td><td></td><td style="background:#c6e0b4"></td><td></td><td></td><td></td><td></td><td></td><td></td></tr>
<tr><td>PDE4DIP</td><td></td><td></td><td></td><td></td><td></td><td></td><td style="background:#c6e0b4"></td><td></td><td></td><td></td><td></td><td></td><td></td></tr>
<tr><td>FXYD1</td><td></td><td></td><td></td><td></td><td></td><td></td><td style="background:#c6e0b4"></td><td></td><td></td><td></td><td></td><td></td><td></td></tr>
<tr><td>STX7</td><td></td><td></td><td></td><td></td><td></td><td></td><td></td><td style="background:#c6e0b4"></td><td></td><td></td><td></td><td></td><td></td></tr>
<tr><td>SIPA1L1</td><td></td><td></td><td></td><td></td><td></td><td></td><td></td><td style="background:#c6e0b4"></td><td></td><td></td><td></td><td></td><td></td></tr>
<tr><td>RNF144B</td><td></td><td></td><td></td><td></td><td></td><td></td><td></td><td style="background:#c6e0b4"></td><td></td><td></td><td></td><td></td><td></td></tr>
<tr><td>KLF5</td><td></td><td></td><td></td><td></td><td></td><td></td><td></td><td></td><td style="background:#c6e0b4"></td><td></td><td></td><td></td><td></td></tr>
<tr><td>ABCA9</td><td></td><td></td><td></td><td></td><td></td><td></td><td></td><td></td><td style="background:#c6e0b4"></td><td></td><td></td><td></td><td></td></tr>
<tr><td>PTGDS</td><td></td><td></td><td></td><td></td><td></td><td></td><td></td><td></td><td style="background:#c6e0b4"></td><td></td><td></td><td></td><td></td></tr>
<tr><td>RASAL2</td><td></td><td></td><td></td><td></td><td></td><td></td><td></td><td></td><td></td><td style="background:#c6e0b4"></td><td></td><td></td><td></td></tr>
<tr><td>ID3</td><td></td><td></td><td></td><td></td><td></td><td></td><td></td><td></td><td></td><td style="background:#c6e0b4"></td><td></td><td></td><td></td></tr>
<tr><td>ETV5</td><td></td><td></td><td></td><td></td><td></td><td></td><td></td><td></td><td></td><td></td><td style="background:#c6e0b4"></td><td></td><td></td></tr>
<tr><td>MAF</td><td></td><td></td><td></td><td></td><td></td><td></td><td></td><td></td><td></td><td></td><td style="background:#c6e0b4"></td><td></td><td></td></tr>
<tr><td>DUSP14</td><td></td><td></td><td></td><td></td><td></td><td></td><td></td><td></td><td></td><td></td><td></td><td style="background:#c6e0b4"></td><td></td></tr>
<tr><td>FAM65B</td><td></td><td></td><td></td><td></td><td></td><td></td><td></td><td></td><td></td><td></td><td></td><td></td><td style="background:#c6e0b4"></td></tr>
</table>
